# Feasibility and preliminary effects of an app-based physical activity intervention for individuals with depression (MoodMover): A protocol for a single-arm, pre-post intervention study

**DOI:** 10.1371/journal.pone.0321958

**Published:** 2025-04-22

**Authors:** Yiling Tang, Madelaine Gierc, Henry La, Sam Liu, Raymond W. Lam, Eli Puterman, Guy Faulkner

**Affiliations:** 1 School of Kinesiology, University of British Columbia, Vancouver, British Columbia, Canada; 2 School of Exercise Science, Physical & Health Education, University of Victoria, Victoria, British Columbia, Canada; 3 Department of Psychiatry, University of British Columbia, Vancouver, British Columbia, Canada; Health Researcher, SPAIN

## Abstract

Depression is the leading cause of disability worldwide. Mobile app-based behavior change interventions that promote lifestyle physical activity (PA) may serve as viable alternatives or adjuncts to traditional treatments offering increased reach and accessibility. This protocol describes an experimental, pre-post single-arm trial to investigate the feasibility and preliminary effects of an app-based, 9-week PA intervention (MoodMover) designed for individuals with depression. MoodMover is co-designed with patients and a multidisciplinary research team using a no-code intervention development platform (Pathverse). This study will employ a single-arm pre-post trial with an optional 9-week follow-up, following the Obesity-Related Behavioral Intervention Trials (ORBIT) model. Thirty-six adults who self-report a clinical diagnosis of major depressive disorder or report at least mild depressive symptoms based on the Patient Health Questionnaire – 9 items (PHQ-9) will be recruited. The main outcomes of this study are the feasibility and acceptability of MoodMover, such as the recruitment strategy, assessments (e.g., PHQ-9), and user engagement. Preliminary effects will be assessed by evaluating changes in PA and depressive symptoms. Recruitment is expected to begin on November 1^st^, 2024, and end on May 1^st^, 2025. Trial results will be disseminated via publications in peer-reviewed journals and via presentations at academic conferences. This study fits within Phase IIa: Proof-of-concept and Phase IIb: Pilot and Preliminary Testing of the ORBIT model. The robust feasibility and acceptability measures, especially the user engagement data powered by Pathverse, will provide a comprehensive understanding of the MoodMover intervention’s feasibility and potential effects. Results will inform potential progression to the next step of the ORBIT model—Phase IIc: Phase II Efficacy Trial—to test MoodMover in a more rigorous randomized controlled trial. This study has been registered at ClinicalTrials.gov (NCT06573125; https://clinicaltrials.gov/study/NCT06573125).

## Introduction

Depression is the leading cause of disability, affecting approximately 280 million people worldwide [1]. Despite a wide range of evidence-based treatments, such as cognitive behavioral therapy and medication, access to adequate care may be limited due to concerns about side effects, resource constraints, a shortage of healthcare professionals, and the stigma associated with seeking mental health services [2]. In Canada, more than half of people seeking mental health care reported unmet needs, particularly for counseling or therapy [3]. Given these barriers, effective alternatives and mental health services with enhanced accessibility are considered a global priority [4].

The positive impact of physical activity (PA) on both preventing and managing depression is well-established [5–7]. Many structured exercise programs have demonstrated moderate antidepressant effects [8], and in Canada, exercise is a recommended first-line treatment for mild-to-moderate depression [9]. However, like psychotherapy, structured exercise interventions often face concerns related to cost and accessibility [10]. Behavior change interventions that promote PA may serve as viable alternatives or adjuncts to conventional treatments, extending their reach to those unable or unwilling to engage in structured exercise programming.

Mobile applications (apps) are increasingly used to deliver behavior change interventions due to their ability to significantly enhance accessibility [11,12]. As of 2013, iTunes and Google Play provided access to a vast selection of over 5,000 apps designed to promote PA [13]. However, these apps have often faced criticism due to the absence of rigorous efficacy testing [13,14], variable use of behavior change techniques [15,16], and were not tailored to people with depression. In particular, our systematic review identified only one randomized controlled trial (RCT) investigating an app-based intervention designed for individuals with depression by November 2021, which demonstrated limited user engagement and efficacy in changing PA [17].

This study will investigate the feasibility and preliminary effects of MoodMover, a 9-week app-based intervention designed to promote PA among people with depression. The development and usability testing of the prototype are described by Tang et al. [18]. This intervention, theoretically grounded in the Multi-Process Action Control (M-PAC) framework [19] for better intention-behavior transition, was developed following the Integrate, Design, Assess, and Share (IDEAS) framework [20] for the enhancement of digital intervention development. The intervention was developed and designed using a no-code app development platform called Pathverse [21] and can be accessed through the Pathverse app (available in both the iOS and Android). The usability of MoodMover was deemed good in our mixed-methods formative study and has been further refined based on feedback from end-users. The revised Obesity-Related Behavioral Intervention Trials (ORBIT) [22] provides a clear path for early intervention development before large efficacy trials. The current study is a part of Phase IIa: Proof-of-concept and Phase IIb: Pilot and Feasibility Testing of the ORBIT model, and we will administer the refined MoodMover in a single-arm, pre-post experimental study. The primary focus of this trial is to assess the feasibility and acceptability of MoodMover, which includes evaluating the recruitment plan, resources (e.g., time and cost), core assessments, intervention fidelity, acceptability of intervention, participant retention, and user engagement. Additionally, we will investigate the preliminary effects of MoodMover in terms of changes in daily step counts as the primary behavioral target. We will also examine changes in depressive symptoms and explore the relationship between these changes.

Five main hypotheses were formulated for the study:

1. The recruitment process will be feasible.
2. PA, as assessed by both smartphone-generated and self-reported data, will show a clinically significant increase (details in Tang et al. [18]) after completing the intervention.
3. Self-reported depressive symptoms will show improvements after completing the intervention.
4. Improvements in the intervention target (i.e., PA) will be associated with positive changes in the clinical outcome (i.e., depressive symptoms).
5. Improvements will be observed in constructs of the M-PAC framework (e.g., affective attitudes).

## Materials and methods

### Study design

This study is an open-label, single-arm pre-post experimental trial with an optional 9-week follow up. The study design, procedures, and data analyses will largely follow those of previous non-randomized feasibility and efficacy studies [e.g., 23–25]. We adhered to the definition of feasibility trials as outlined in the 2010 Consolidated Standards of Reporting Trials (CONSORT) guidelines [22,26]. This non-randomized pre-post study with the primary aim of evaluating feasibility will inform future RCTs [27]. In addition, we will evaluate preliminary effects of the intervention and report estimations, regarding the behavioural outcome. The reporting of this protocol follows a guide proposed by Thabane and Lancaster [28], recommending the use of the SPIRIT (Standard Protocol Items: Recommendations for Interventional Trials) checklist [29], supplemented by items from the CONSORT 2010 statement (see S1 File). The final study will follow a guide proposed by Lancaster and Thabane [30], recommending the use of the STROBE (Strengthening the Reporting of Observational Studies in Epidemiology) statement alongside with the CONSORT extension for pilot and feasibility trials. Ethical approval (see the complete study protocol in S2 File) is obtained from the University of British Columbia Behavioural Research Ethics Board (BREB) (H24-01820). Any substantive protocol modifications (e.g., amendments that can affect study validity) [29] will be formally reviewed and approved by the Research Ethics Boards. A list of amendments will be transparently reported in the final study. This trial is registered at ClinicalTrials.gov (NCT06573125).

### Participation eligibility

This study will include outpatients aged 18–64 years who self-report a diagnosis of major depressive disorder or self-report at least mild depressive symptoms, as indicated by scoring at least 5 on the 9-item Patient Health Questionnaire (PHQ-9) [31]. Participants will be required to demonstrate literacy in English, possess an active email address, and own either an iPhone or an Android smartphone with internet capability to download the Pathverse app to access the MoodMover program. This study will include individuals who self-report engaging in less than 90 minutes of moderate-vigorous PA per week. Concomitant care and interventions will be allowed during the trial; participants may continue using medications provided the dosage remains unchanged. All participants must give fully informed consent on REDCap [32]. Exclusion criteria include self-reporting of a physical disability and/or health condition that prevents exercise (e.g., unstable angina, uncontrolled diabetes, acute heart failure), active psychosis or mania, active suicidal thoughts, severe cognitive impairment (e.g., major neurocognitive disorder), and/or being currently pregnant. In addition, individuals will not be eligible if they anticipate a major absence (e.g., vacation, surgery) in the next three months.

### Recruitment

Patients will be recruited using multiple strategies. First, a brief introduction to this research study will be posted on REACH BC [33] and other research institute websites in Canada (e.g., [34]). Second, UBC mental health counseling services and other healthcare providers across Canada, as well as free depression advocacy groups (potential groups can be found at [35,36]), will be approached for potential dissemination of study information. Third, posters will be placed in different hospitals and clinics in British Columbia, Canada. Notably, remote recruitment will be employed to mimic real-world conditions, and not all recruitment strategies will be implemented simultaneously. The selection and modification of recruitment strategies will consider available resources, time constraints, and the speed of recruitment. Potential participants will complete a screening questionnaire on REDCap (see S3 File). Individuals who are found ineligible due to active suicidal thoughts will be informed immediately via email, and they will be provided with information about other community resources available for mental health support. Those who meet the eligibility criteria will be invited to participate in the study. Interested and eligible individuals will receive detailed study information via email and will complete the informed consent process electronically and all baseline measures of interest on REDCap. Due to remote recruitment, only electronic signatures will be obtained, as approved by the ethics committee. Potential participants will be given one week to consider the provided information, and they will receive a second invitation to confirm their participation through email. Participants can withdraw from the treatment or the study at any time.

### Procedures

After an electronical consent form and baseline measures are completed, a researcher will register participants to the MoodMover program on the Pathverse admin web portal using their provided email address. Participants will receive an email with a MoodMover user guide, an instructional letter including information about downloading the Pathverse app from either the Apple Store or Google Play, and how to share their smartphone screens over Zoom. In particular, they will be instructed to sign up and log into Pathverse using the email address they provided.

Participants will be required to take a 15-minute one-on-one orientation session via Zoom over their smartphones. Before the orientation, participants will be asked to read through the MoodMover user guide. During the orientation, participants will share their smartphone screens and complete the first introduction module themselves while the researcher observes and provides explanations as needed, particularly regarding the ramped step goal recommendations. Following the completion of the first module, the researcher will hold a brief Q&A session to address any remaining questions or concerns the participant may have. Detailed scripts will be used to ensure consistent delivery of information across participants. Participants will be asked if they consent to having their session recorded at the start of their orientation and will be asked to verbally confirm their consent at the beginning of the recording. Only five orientation sessions will be recorded and assessed by a research assistant using a fidelity checklist after each session, which will allow immediate modifications to improve treatment fidelity [37]. The start date of the program will be set as the day of the orientation session. Participants can postpone the start date if needed by informing the researcher via email.

**Fig 1:**
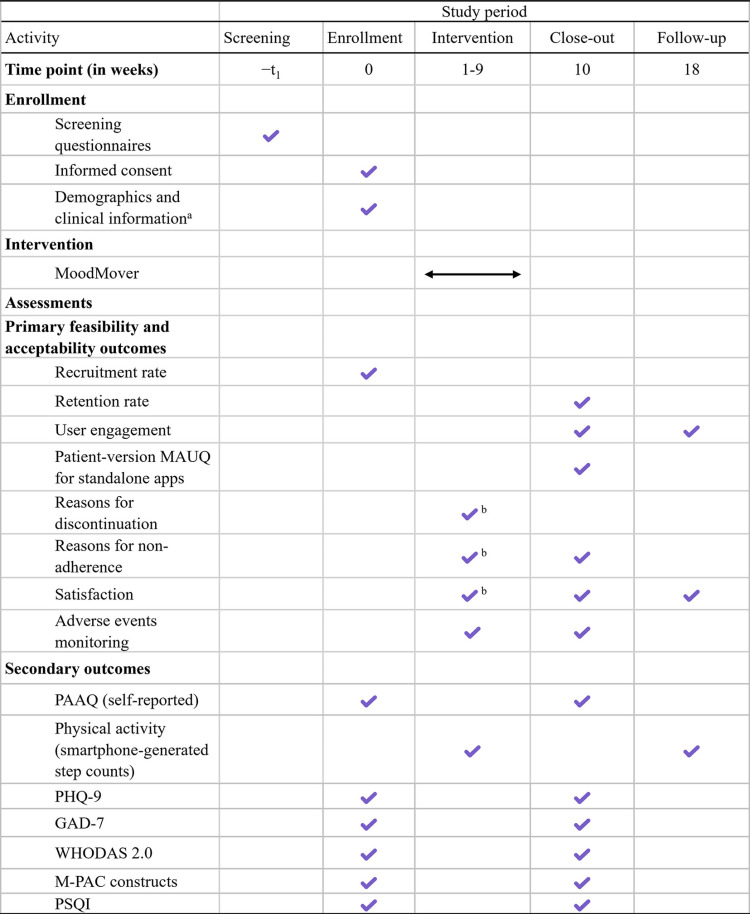
The schedule of enrollment, interventions, and assessments. Note. GAD-7: Generalized Anxiety Disorder 7; MAUQ: mHealth App Usability Questionnaire; M-PAC: Multi-Process Action Control; PAAQ: Physical Activity Adult Questionnaire; PHQ-9: Patient Health Questionnaire – 9 items; PSQI: Pittsburgh Sleep Quality Index; WHODAS 2.0: World Health Organization Disability Assessment Schedule 2.0. ^a^Includes: sex, gender, race/ethnicity, age, height, weight, the highest level of education, employment status, income, disease duration, medication use, other ongoing treatments relevant to depression management, substance use, and current and past usage of physical activity apps or devices. ^b^For early discontinuation.

After completing the intervention, participants will undergo a re-assessment on REDCap. Participants will be encouraged to keep the app on their smartphones and continue using the app after completing the program. Choosing not to uninstall the app will be considered as providing consent, allowing the researcher to access and track their user engagement for a follow-up at 9 weeks. These participants will be approached on the last day of the follow-up period to sync their steps with MoodMover again, allowing the researcher to collect their PA data during the entire follow-up period. In addition, participants will be asked to complete a satisfaction questionnaire on REDCap. All study participants will be compensated $10 or gifts of equivalent value for completing each pre- and post-intervention assessment. The enrollment, intervention, and assessment schedule, following the recommended format by the SPIRIT guidelines, aligning with relevant items from the CONSORT 2010 guidelines [26], is presented in Fig 1.

### Intervention

MoodMover is a 9-week app-based intervention designed to increase PA among people with depression delivered through Pathverse [18]. The content was contextualized for people with depression based on an existing 10-week, web-based PA intervention for young adults guided by the M-PAC framework [23]. The current version of MoodMover consists of one run-in module for introduction and baseline self-monitoring, eight major modules, and eight complementary (optional) modules. After completing the first week, participants will be encouraged to set daily step goals by increasing an additional 1000 steps above their baseline PA level (week 1) every two weeks until reaching an increment of 3000 steps per day [18,38,39]. S4 File depicts each major lesson’s topics. A combination of different formats has been employed for delivering educational content, including videos, podcasts, and illustrated articles.

**Fig 2:**
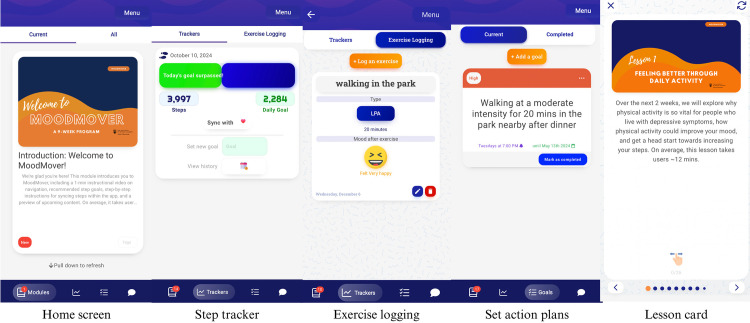
Screenshots of home screen, step tracker, exercise logging, action planning, and a card of a major lesson (for illustrative purposes only).

**Fig 3:**
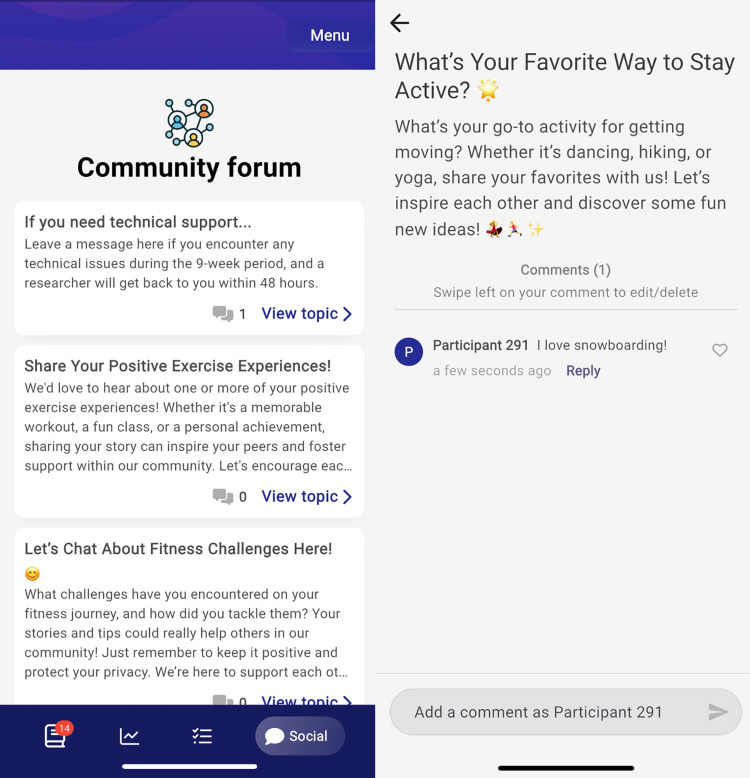
Screenshots of the peer-to-peer communication forum.

The app incorporated a wide range of behavioral change techniques (BCTs; e.g., self-monitoring and graded tasks) following the BCT taxonomy V1 [40] to implement the behavioral constructs of the M-PAC model. Apart from structured psychoeducational content, the app also offers diverse behavioral features, allowing participants to set step goals and action plans, monitor their progress, and engage in anonymous communication with other participants on pre-determined topics related to exercising. To improve engagement and promote PA, MoodMover also incorporated brief notifications and a gamification element where participants can earn 20 points for completing each major lesson and 10 points for completing each complementary lesson. Every 60 points earned is equivalent to a $5 CAD e-gift card. Screenshots in Fig 2 illustrate the home screen, step tracker, exercise log (with mood monitoring after exercising), and a card of a major lesson of the refined prototype of MoodMover. Screenshots in Fig 3 illustrate the community forum of MoodMover. The program details are described elsewhere (Tang et al. [18]). Participants will be instructed to read the lessons and complete all activities within each lesson over a 9-week period, with one lesson (week 1) and two lessons (one major and one complementary lesson; week 2–9) released at pre-scheduled times per week.

### Measures

#### Demographic and clinical information.

All participants will be asked to provide demographic and clinical information (see S5 File), such as sex and gender, race/ethnicity, age, height, weight, highest level of education achieved, employment status, income, duration of depression, medication use, other ongoing treatments relevant to depression management, substance use (e.g., cannabis), and current and past usage of physical activity apps or devices.

#### Primary feasibility and acceptability outcomes.

The traffic light system will be used to determine whether proceeding to a RCT based on the worst-performing feasibility criterion [41]. The 3-tiered progression criteria require setting cut-offs for green (proceed without changes), amber (proceed with amendments), and red (stop proceeding) zones for each of the key feasibility criterion. Table 1 presents the pre-determined thresholds for recruitment, adherence, usability, and retention based on previous literature, our usability testing study of MoodMover [18], pragmatic considerations, and expert insight within the research team. In addition, compliance to the graded goal setting and user satisfaction will be assessed to support the interpretation of feasibility and acceptability.

**Table 1:** Pre-determined progression criteria.

Feasibility outcomes	Green zone (go)	Amber zone (amend)	Red zone (stop)
**Recruitment**	≥65% of eligible participants	40%- <65% of eligible participants	<40% of eligible participants
**Adherence**	≥70% of participants complete the minimum requirements, that is, completing 5 major lessons	40%- <70% of participants complete the minimum requirements, that is, completing 5 major lessons	<40% of participants complete the minimum requirements, that is, completing 5 major lessons
**Retention**	≥70% participants complete the post-intervention assessment	40%-<70% participants complete the post-intervention assessment	<40% participants complete the post-intervention assessment
**Usability**	≥70% of participants scored 5 and over on MAUQ	40%- <70% of participants scored 5 and over on MAUQ	<40% of participants scored 5 and over on MAUQ

*Note.* MAUQ: mHealth App Usability Questionnaire.

Recruitment*.* Recruitment rate will be calculated by dividing the number of people with depression who enrolled in the trial by the number of interested individuals meeting the eligibility criteria. Successful recruitment will be defined as achieving a rate of 65%. This progression criteria was based on studies with similar feasibility-based designs and those sampling from adults with mental illness [e.g., 42]. The time taken, source of recruitment, and the number of recruitment sites (if placed posters) to recruit the targeted sample size will also be recorded.

Adherence. Intervention adherence will be based on the proportion of participants who complete the majority (five out of eight) of major lessons. Previous systematic reviews identified a wide range of adherence rates for mHealth apps among people with depression. For example, 2/3 of studies in Serrano-Ripoll et al. [43] demonstrated a adherence rate of less than 50% (if reported). In contrast, Kerst et al. [44] observed adherence rates ranging from 70% to 94% in the majority (5/7) of the reviewed studies; however, remarkably lower adherence rates (22% and 35%) in two studies. In this study, an adherence rate of 70% will be deemed as falling within the green zone. Other user engagement metric data (i.e., time spent on each module) will be also downloaded from the Pathverse admin web portal. Various available objective engagement metrics, as summarized in Molloy and Anderson [45], will be calculated and reported, including use of specific program features (e.g., number of times participants changed their step goals, number of action plans completed, and number of exercise sessions logged), program use by the number of active days (i.e., number of days the program is used at least once), total duration of use, and average duration for completing the major and complementary modules. Participants who complete all 8 major modules will be categorized as “complete users”; those who complete 2–8 modules will be categorized as “incomplete users”; and those who do not use the app after the orientation session will be categorized as “nonusers”, respectively. Ongoing monitoring of adherence will be employed. Participants who have not opened the app for two weeks will be contacted for a check-in via email. Reasons for non-adherence (if provided) will be recorded.

Usability*.* The adapted, patient version of the mHealth app usability questionnaire (MAUQ) [46] designed for standalone apps will be employed. The MUAQ contains 13 items (see S6 File and Tang et al. [18]). It evaluates three domains of usability as defined by the International Organization for Standardization (ISO) definition of usability [47]: ease of use (MAUQ_E; 5 items, e.g., “The app was easy to use”), usefulness (MAUQ_U; 1 item, e.g., “The app would be useful for my mental health and well-being”), and interface and satisfaction (MAUQ_I; 7 items, e.g., “The information in the app was well organized, so I could easily find the information I needed.”). Participants will be asked to rate each item on a 7-point Likert scale (1 = strongly disagree to 7 = strongly agree). An average score of 5 and over will be deemed as acceptable consistent with Tang et al. [18].

Retention rates. Retention will be determined by the proportion of participants completing both pre- and post-intervention questionnaires. Torous et al. [48] found an average attrition rate of 26.2%, rising to 47.8% when adjusted for publication bias, among mental health apps for depression, with lower rates observed in those with mood tracking (18.4%) and human feedback (11.7%). An attrition rate of 30% will be defined as acceptable (green zone) for MoodMover. Additionally, reasons for discontinuation and any adverse events will be recorded.

Compliance. The compliance rate to the graded goal setting will be reported. Specifically, the proportion of participants who successfully set their step goal in week 2, and those who modified their step goals in week 4 and week 6, will be reported separately. Participants who followed all the instructions across these time points will be deemed as having achieved “full compliance”, while those who followed instructions at one or two time points will be deemed as having achieved “partial compliance”. Participants who did not follow instructions at any time point will be deemed as having achieved “no compliance”.

Satisfaction. Users’ satisfaction levels will be evaluated using Melin et al.’s mHealth Satisfaction Questionnaire [49] at both post-intervention and the 9-week follow-up (optional). This 14-item questionnaire is recommended by Hajesmaeel et al. [50] as it was specifically designed for mHealth applications (see S7 File). Participants will be asked to rate on a 5-point Likert scale (1 = strongly disagree to 5 = strongly agree). Among 14 items, four items are negatively stated and will be reversed in the analyses. The total score ranges from 14 to 60, with a higher score indicating a higher satisfaction level. In addition, participants will be asked to list three things they liked and disliked about the intervention.

#### Secondary outcomes.

While this study is not powered to determine the intervention’s efficacy definitively, we will still collect data on each outcome to investigate interval estimates of the changes. The response rate and completion rate of each measure will be reported to inform the feasibility of administering the selected measures in a future trial. Additionally, we aim to assess the potential of MoodMover to produce clinically significant PA and depression changes in a larger evaluation.

Physical activity (behavioral outcome)*.* Change in daily step counts will be collected using the smartphone’s built-in step counting function, which will be synced with MoodMover and can be downloaded from the Pathverse admin web portal. A significant increase of 3000 daily steps for 5 days per week will be deemed as reaching the clinically significant behavioral goal of MoodMover [18]. Moreover, to evaluate self-reported PA, we will utilize the Canadian Physical Activity Adult Questionnaire (PAAQ) [51]. This questionnaire is designed in accordance with the Canadian Physical Activity Guidelines, which recommend a minimum of 150 minutes of MVPA per week for adults (see S8 File). It has been widely employed for monitoring PA in Canadian adults and has demonstrated a stronger association with accelerometer-based data than the International Physical Activity Questionnaire [51,52]. Along with the PAAQ questionnaire, participants will be asked at post-intervention whether they have linked a smartwatch or fitness device to Health (iOS) or Google Fit (Android) throughout the program. Additionally, they will be asked how often (almost always, sometimes, seldom) they carried their phone (or wore their smartwatch, if used) during their non-sedentary waking hours during workdays and weekend (“How frequently do you carry your phone/wear your smartwatch with you during your non-sedentary waking hours?”).

Depressive symptoms (clinical outcome)*.* Depression severity will be evaluated utilizing the PHQ-9 [31], a widely used self-report scale that demonstrates sensitivity in detecting changes after treatment in psychiatric patients [53]. Participants will be asked to rate 9 items, using 0-to-3 Likert scales, resulting in a total score ranging from 0 to 27 (see S3 File). Higher scores reflect more severe depressive symptoms. The classification of depression severity will be as follows: a score of 5–9 will be considered indicative of mild depressive symptoms, 10–14 as moderate, 15–19 as moderately severe, and 20–27 as severe depressive symptoms [54]. To define a clinically meaningful change in depression, McMillan et al. [55] proposed a reduction of 5 points on the PHQ-9, along with a PHQ-9 score shift from ≥ 10 at baseline to ≤ 9 at post-intervention to be considered clinically relevant. Given that individuals with mild depressive symptoms will be eligible for the current study, this classification will be exclusively applied to those who scored 10 or higher at baseline.

Anxiety. Anxiety will be measured by the Generalized Anxiety Disorder 7 scale (GAD-7) [56] (See S9 File). The GAD-7 is a seven-item validated [57] instrument for measuring the severity of anxiety on four-point Likert scales (0 = “not at all” to 3 = “nearly every day”). The total score on the scale ranges from 0 to 21, with higher scores indicating severe anxiety. The classification of anxiety severity will be as follows: 5 for mild, 10 for moderate and 15 for severe [57].

Sleep quality. The Pittsburgh Sleep Quality Index (PSQI) [58] is a self-rated, 19-item questionnaire with strong reliability and validity [59] that assesses various sleep-related problems over the past month (see S10 File). The 19 questions are categorized into seven components, including sleep quality, sleep duration, sleep latency, habitual sleep efficiency, sleep disorders, use of sleep medication, and daytime dysfunction. Participants will be asked to rate on a 4-point Likert scale ranging from 0 to 3. A total score ranging from 0 to 21 can be summed for these seven components, with a higher score reflecting poorer sleep quality. A total score of >5 indicates a poor quality of sleep.

Functional disability. World Health Organization Disability Assessment Schedule 2.0 (WHODAS 2.0) [60] will be utilized to evaluate participants’ health and ability to perform activities in six areas of functionality: cognition, mobility, self-care, getting along, life activities, and participation (See S11 File). The WHODAS 2.0 comprises 12 items. For each item, participants will be asked to estimate the extent of their disability over the past 30 days, using a 5-point Likert scale ranging from 1 (none) to 5 (extreme/cannot do). The total score on the scale ranges from 0 to 100, with higher scores indicating a greater degree of disability. WHODAS 2.0 has good internal consistency across all domains (α =.77 -.98) [61].

#### M-PAC constructs.

The measures of the reflective and regulatory processes within the M-PAC framework will be primarily derived from Tang et al. [62]. The M-PAC survey is presented in S12 File.

Reflective processes*. Affective attitudes* and *instrumental attitudes* will be measured on three 7-point bipolar scales, respectively. All items share the same stem: “For me, participating in regular PA over the next month would be...” Higher scores indicate more positive attitudes. The items had demonstrated very good to excellent inter-item reliability in Tang et al. [62] (affective attitudes, α =.87; instrumental attitudes, α =.91). *Perceived opportunity* and *perceived capability* will be assessed using three 5-point Likert scales, ranging from 1 (strongly disagree) to 5 (strongly agree), respectively. The items have shown acceptable inter-item reliability (opportunity, α =.70; capability, α =.67) [63].

Intention. *Intention strength* will be measured using three items (e.g., I am committed to engage in physical activity over the next month). Participants will rate these items on 5-point Likert scales ranging from 1 (strongly disagree) to 5 (strongly agree). The items have exhibited very good inter-item reliability (α =.89) [62]. In addition, *decisional intention* will be evaluated using one item regarding the intention to perform more PA and achieve the behavioral outcome goal of increasing 3000 daily steps above their baseline PA levels for most days per week. Participants will be asked to provide binary responses (Yes/No).

Regulatory processes*. Regulations* will be measured using 5-point Likert scales with three items pertaining to self-monitoring, goal-setting, and action planning. The inter-item reliability for the regulatory process measures has been found to be very good (α =.81) [62].

Reflexive processes. *Habit* will be assessed using three items (e.g., “I engage in regular physical activity without having to consciously remember it”), which have been modified from Rhodes and Lim [64] and were originally derived from the subscale of the Self-Report Habit Index [65]. *Identity* will be measured using three items (e.g., “When I describe myself to others, I usually include my involvement in physical activity”), which have been adapted from previous studies [64,66]. Both measures will utilize a 5-point Likert scale ranging from 1 (strongly disagree) to 5 (strongly agree) for participant responses. The inter-item reliability of the habit measure has demonstrated excellent internal consistency (α =.93), while the identity measure has shown very good internal consistency (α =.87) [64].

### Sample size justification

As a single-arm feasibility trial, the ideal sample size was informed by the recommendations for the intervention arm of pilot RCTs, based on the “traffic light” progression criteria [67]. The traffic light system proposed by Lewis et al. [67] is regarded as a recent and robust approach for sample size justification in feasibility and pilot studies and has been recommended for application in the physical activity domain[68]. Four feasibility criteria (i.e., recruitment, adherence, usability, and retention) were considered individually (see Table 1). To achieve over 90% statistical power for each criterion, as aligned with the quick look-up grid for sample size based on the normal approximation approach in Lewis et al. [67], the study requires: (1) 36 participants for recruitment; (2) 25 participants in the intervention group to assess adherence; (3) 25 participants in the intervention group to assess usability; and (4) 25 participants for retention. The overall sample size was determined by the criterion with the highest requirement—adherence and usability, requiring 25 participants per arm. Considering a 30% attrition rate, the sample size for adherence increases to 36 participants. With an expected 65% recruitment success as our previous usability testing study indicated, 55 eligible patients need be invited to achieve the target sample size (55 = 1/0.65 × 36). Considering the available resources and time, a pragmatic sample size range of 20–36 will be adopted, incorporating commonly accepted sample size rule of thumbs. Julious et al. [69] suggests a minimum of 12 participants per arm, whereas Kieser and Wassmer [70] recommended a range of 10–20 per arm for pilot studies.

### Data management

During data collection, all questionnaire data will be collected and collated on the UBC-hosted data management platform, REDCap. App-related data will be collected by Pathverse and stored in Canada on Amazon Web Services (AWS) [71]. Five orientation sessions will be transcribed using Otter.ai [72], which stores data in the AWS West region, United States. After the intervention, all data will be downloaded and securely stored on a UBC-secured, password-protected computer, and then transferred to UBC’s secure OneDrive. All files will be password-protected and encrypted. Only authorized researchers involved in the study will have access to the data. After data collection is completed, any personal identifying information will be deleted. As is common practice in scientific research, other researchers may access to the de-identified data after publications. Participants will be fully informed of these procedures before consenting to this trial.

### Statistical analysis

All analyses will be conducted using either SPSS ver. 29 or R (version 4.2.2) via RStudio (version 2024.12.0+467). Descriptive statistics will be presented for all measures. Missing data in feasibility variables and smartphone-based step counts will be reported and interpreted as part of the feasibility results. In a specific case of missing smartphone-based step data in the final week (week 9), the last observation carried forward method will be used to substitute missing data with values from week 8 for preliminary effects related analyses, as both weeks fall within the PA maintenance phase. The strategy for addressing missing data in other secondary outcomes will be determined by evaluating the degree and pattern of missingness, i.e., Missing Completely at Random (MCAR), Missing at Random (MAR), Missing Not at Random (MNAR) [73]. For example, Little’s MCAR tests [74] will be conducted using SPSS. If the data are determined to be MCAR or MAR, mean substitution will be applied when the amount of missing data is minimal (<5–10%). Pairwise deletion will be employed when there is moderate missing data (10–20%). In cases of a substantial amount of missing data (>20%) or if the missing data are determined to be MNAR, no further analyses will be conducted to avoid biased estimates. The impact of potentially influential factors (e.g., depression severity and age) on feasibility results (e.g., lesson completion), will be explored using regression-based analyses. A separate regression model will be run for each factor. Regression analyses will be conducted only if at least 10 participants per variable provide valid data, adhering to established rules of thumb for regression analyses [75]. Additionally, we will explore the changes in secondary outcomes (e.g., PA, depression, and M-PAC constructs) over time. Given the small sample size and two time points (pre- and post-intervention), paired t-tests will be used for continuous outcomes, while equivalent non-parametric tests (i.e., Wilcoxon signed-rank tests) will be applied for non-normal distributions. For binary outcomes, McNemar’s test will be employed. The association between changes in PA and changes in depressive symptoms will be assessed using Pearson’s or Spearman’s correlation analysis. As an underpowered single-arm feasibility study, the reporting will focus on estimation. Results will be reported with 75%, 85%, and 95% confidence intervals, while considering clinically meaningful changes as suggested by Lee et al [76] and Walters [77]. A binary variable will be generated to indicate whether a participant met the behavioral step goal (1) or not (0). Specifically, the pre-determined clinically meaningful decrease in depression will be incorporated into the interpretation of the results for those who scored 10 or above on the PHQ-9 at baseline.

### Harms and data monitoring

The research protocol described here is categorized as low research risk and low to moderate participant vulnerability. The intervention is not anticipated to cause physical harm. The app focuses on promoting lifestyle physical activities, which carry no greater risk than participants would experience in their daily lives. Although using an app for data collection introduces moderate privacy and confidentiality risks, the Pathverse platform employs strong technical standards and encryption. Access to the Pathverse web portal is password-protected, and all collected data is deidentified and encrypted during transit and storage. Therefore, privacy and confidentiality risks are considered low. Participants, who may have varying levels of depression or other mental health issues, represent a vulnerable group. They might experience a change in their mental health status inherently or emotional distress if unable to meet daily step goals. As a remote study, researchers will not be able to provide in-person and/or immediate support for participants, placing this risk in the moderate range. However, concomitant care is permitted, including stable medication use, contributing to a low to moderate level of participant vulnerability. Additionally, participants will be provided with information on 24/7 mental health supports (e.g., national crisis lines) in case of urgent need. As this trial has a short duration (9 weeks) and generally low-risk considerations, a data monitoring committee is not incorporated.

### Timeline and dissemination plans

Recruitment is anticipated to begin on November 1^st^, 2024. While efforts will be made to achieve the ideal sample size, recruitment will conclude at the 6-month mark from the start of data collection (estimated to be May 1, 2025) at the latest if the minimum sample size is reached. Trial results will be disseminated via publications in peer-reviewed journals and via presentations at academic conferences.

## Discussion

To provide greater evidence-based treatment options for patients with depression who are not interested in, or have no access to conventional depression treatments, well-designed app-based intervention that can increase PA and sustain those increases over time may be beneficial. This trial will explore the feasibility, usability, and acceptability of a 9-week, theory-based (M-PAC) app designed for people with depression following a combination of two systematic and rigorous behavioral intervention development frameworks (IDEAS and ORBIT). This approach is crucial for refining the intervention, improving study procedures, and informing the design of future definitive studies [26]. In addition, we will explore the potential effect of MoodMover on participants’ behavioral (PA) and clinical outcomes (e.g., depression). We hypothesize that this intervention will demonstrate potential in increasing multiple M-PAC constructs, such as affective attitudes, regulatory skills, habit, and identity. Both habit and identity are powerful theoretical constructs that we hope can produce sustained, long-term change in exercise behavior patterns.

One strength of this study is that the current version of MoodMover has passed usability testing among the target population and has been refined based on participant feedback [18]. In addition, MoodMover was designed based on the M-PAC framework, a behaviour change framework that has been deemed suitable for people with poor mental health [62], and incorporated a series of BCTs corresponding to the M-PAC constructs, along with additional BCTs to enhance engagement. Moreover, this study will include a more robust analysis of user engagement data powered by the Pathverse platform, whereas most previous studies with mHealth apps for depression reported only one or two metrics [45]. Researchers can closely track PA behavior (i.e., daily step counts) and the timing and reasons for app discontinuation, shedding light on the app usage lifecycle. In addition, an extensive range of potential influential factors will be assessed, aiding in the understanding of the associations between engagement and other variables. Understanding how app use changes over extended periods of time, and assessing various mediating variables and process measures, will provide insights into the mechanisms behind the interventions’ success or failure and inform either moving to a larger efficacy trial, moving backwards to the previous phases of the IDEAS model for refinement, or abandoning further development of MoodMover.

A potential limitation is the common high dropout rates and low user engagement often faced by mHealth apps for depression [48] which could impact the evaluation of MoodMover’s preliminary effects. Torous et al. [48] found that apps incorporating mood monitoring showed a considerably lower dropout rate of 18.4%. Given this, we have incorporated a mood monitoring element by asking participants to rate their mood after exercising when they log their exercise behaviors. Moreover, to enhance the likelihood of sustained app usage, we have introduced several evidence-based elements: a gamification feature with incentives, a peer-to-peer support forum, reminders/notifications, an easy-to-use interface, and a combination of multiple formats to deliver educational content (see Tang et al. [18]). We anticipate that these strategies will increase adherence and engagement while potentially improving MoodMover’s preliminary effects in promoting PA. The exit satisfaction survey will provide feedback on the features of MoodMover perceived as being most and least helpful. In addition, the inclusion criteria for this feasibility study—including a broad age range, a spectrum of depression severity (from mild to clinically significant), and the allowance for concurrent clinical care and medication use—were intentionally designed to reflect real-world conditions. However, due to the small sample size, this heterogeneity may limit the interpretability of the feasibility outcomes. Large-scale future studies may be warranted to examine subgroup differences through stratified analyses.

Overall, this study fits within Phase IIa and Phase IIb of the ORBIT model. The present study will provide insights into the potential of MoodMover serving as an alternative strategy or adjunct treatment for managing mild-to-moderate depression, which can be implemented into clinical mental health care in Canada. Despite the possibility of null effects, this study will yield valuable data related to recruitment plans, trial processes and procedures (e.g., core assessments and app features), resources, trial management, and other feasibility variables (e.g., user engagement and satisfaction). In contrast, promising results will allow us to move to the next step, Phase IIc: Phase II Efficacy Trial, of the ORBIT model for testing the efficacy of MoodMover on behavioural outcome in an appropriately powered RCT. Other study designs, such as sequential, multiple assignment, randomized trials (SMARTs) [78], micro-randomized trials [79], single-case designs [80], dose-finding methods, may also be considered for assessing MoodMover in the future.

## Supporting information

S1 File The SPIRIT checklist. (DOCX)

S2 File The study protocol. (DOCX)

S3 File Screening questionnaire. (DOCX)

S4 File Topics of major lessons. (DOCX)

S5 File Demographics and clinical information. (DOCX)

S6 File Adapted MAUQ – Patient version for standalone apps. (DOCX)

S7 File Melin’s mHealth Satisfaction Questionnaire. (DOCX)

S8 File Physical Activity Adult Questionnaire. (DOCX)

S9 File GAD-7. (DOCX)

S10 File Pittsburgh Sleep Quality Index (PSQI). (DOCX)

S11 File WHODAS2.0–12items. (DOCX)

S12 File M-PAC constructs. (DOCX)
