# Supplementary material for: Feasibility and preliminary effects of an app-based physical activity intervention for individuals with depression (MoodMover): A protocol for a single-arm, pre-post intervention study
Source: PLoS One. 2025 Apr 22;20(4):e0321958. doi: 10.1371/journal.pone.0321958 (PMC12013873; doi:10.1371/journal.pone.0321958)
Supplement: S2 File — (DOCX) [file pone.0321958.s002.docx]

**Feasibility and preliminary efficacy of an app-based physical activity intervention for individuals with depression (MoodMover): A protocol for a single-arm, pre-post intervention study**

Yiling Tang^1*^, Madelaine Gierc^1^, Henry La^2^, Sam Liu^2^, Raymond W Lam^3^, Eli Puterman^1^ & Guy Faulkner^1^

**Abstract**

Background:

Depression is the leading cause of disability worldwide. Mobile app-based behavior change interventions that promote lifestyle physical activity (PA) may serve as viable alternatives or adjuncts to traditional treatments offering increased reach and accessibility.

Objectives:

This protocol describes an experimental, pre-post single-arm trial to investigate the feasibility and preliminary efficacy of an app-based, 9-week PA intervention (MoodMover) designed for individuals with depression.

Methods:

MoodMover is a 9-week intervention co-designed with patients and a multidisciplinary research team, following the Integrate, Design, Assess, and Share (IDEAS) framework, theoretically grounded in the Multi-Process Action Control (M-PAC) framework, and using a no-code intervention development platform (Pathverse). This study will employ a single-arm pre-post trial with a 9-week follow-up, following the Obesity-Related Behavioral Intervention Trials (ORBIT) model, which provides a clearer path for early behavioural intervention development before large efficacy trials. Thirty-six adults with depression or at least mild depressive symptoms will be recruited. The main outcomes of this study are the feasibility and acceptability remof MoodMover. This includes examining the recruitment strategy, resource requirements, assessments (e.g., Patient Health Questionnaire – 9 items), intervention fidelity, acceptability of intervention, participant retention, and user engagement. Preliminary efficacy will be assessed by evaluating changes in PA and depressive symptoms.

Results:

Development and usability testing of MoodMover has been completed. Ethics approval for this pilot study has been obtained from Research Ethics Boards at the University of British Columbia (H24-01820). Recruitment is expected to begin October 2024; data collection and analysis are expected to be completed by June 2025. Trial results will be disseminated via publications in peer-reviewed journals and via presentations at academic conferences.

Conclusions:

This study fits within Phase IIa: Proof-of-concept and Phase IIb: Pilot and preliminary Testing of the ORBIT model. The robust feasibility and acceptability measures, especially the user engagement data powered by Pathverse, will provide a comprehensive understanding of the MoodMover intervention's feasibility and potential efficacy. Results will inform potential progression to the next step of the ORBIT model—Phase IIc: Phase II Efficacy Trial—to test MoodMover in a more rigorous RCT.

Trial registration:

ClinicalTrials.gov NCT06573125; <https://clinicaltrials.gov/study/NCT06573125>

Keywords

mHealth; mobile health; app; feasibility; randomized controlled trial; depression; mental health; pilot

## Introduction

Depression is the leading cause of disability, affecting approximately 280 million people worldwide [1]. Despite a wide range of evidence-based treatments, such as cognitive behavioral therapy and medication, access to adequate care may be limited due to concerns about side effects, resource constraints, a shortage of healthcare professionals, and the stigma associated with seeking mental health services [2]. In Canada, more than half of people seeking mental health care reported unmet needs, particularly for counseling or therapy [3]. Given these barriers, effective alternatives and mental health services with enhanced accessibility are considered a global priority [4].

The positive impact of physical activity (PA) on both preventing and managing depression is well-established [5-7]. Many structured exercise programs have demonstrated moderate antidepressant effects [8], and in Canada, exercise is a recommended first-line treatment for mild-to-moderate depression [9]. However, like psychotherapy, structured exercise interventions often face concerns related to cost and accessibility [10]. Behavior change interventions that promote PA may serve as viable alternatives or adjuncts to conventional treatments, extending their reach to those unable or unwilling to engage in structured exercise programming.

Mobile applications (apps) are increasingly used to deliver behavior change interventions due to their ability to significantly enhance accessibility [11, 12]. As of 2013, iTunes and Google Play provided access to a vast selection of over 5,000 apps designed to promote PA [13]. However, these apps have often faced criticism due to the absence of rigorous efficacy testing [13, 14], variable use of behavior change techniques [15, 16], and were not tailored to people with depression. In particular, our systematic review identified only one randomized controlled trial (RCT) investigating an app-based intervention designed for individuals with depression by November 2021, which demonstrated limited user engagement and efficacy in PA promotion [17].

This study will investigate the feasibility and preliminary efficacy of MoodMover, a 9-week app-based intervention designed to promote PA among people with depression. The development and usability testing of the prototype are described by Tang et al. [18]. This intervention, theoretically grounded in the Multi-Process Action Control (M-PAC) framework [19] for better intention-behavior transition, was developed following the Integrate, Design, Assess, and Share (IDEAS) framework [20] for the enhancement of digital intervention development. The intervention was developed and designed using a no-code app development platform called Pathverse [21] and can be accessed through the Pathverse app (available in both the iOS and Android). The usability of MoodMover was deemed good in our mixed-methods formative study and has been further refined based on feedback from end-users. The revised Obesity-Related Behavioral Intervention Trials (ORBIT) [22] provides a clear path for early intervention development before large efficacy trials. The current study is a part of Phase IIa: Proof-of-concept and Phase IIb: Pilot and Feasibility Testing of the ORBIT model, and we will administer the refined MoodMover in a single-arm, pre-post experimental study. The primary focus of this trial is to assess the feasibility and acceptability of MoodMover, which includes evaluating the recruitment plan, resources (e.g., time and cost), core assessments, intervention fidelity, acceptability of intervention, participant retention, and user engagement. Additionally, we will investigate the preliminary efficacy of MoodMover in terms of changes in daily step counts as the primary behavioral target. We will also examine changes in depressive symptoms and explore the relationship between these changes.

Five main hypotheses were formulated for the study:

1. The recruitment process will be feasible.
2. PA, as assessed by both smartphone-generated and self-reported data, will show a clinically significant increase (details in Tang et al. [18]) after completing the intervention.
3. Self-reported depressive symptoms may show improvements after completing the intervention.
4. Improvements in the intervention target (i.e., PA) will be associated with positive changes in the clinical outcome (i.e., depressive symptoms).
5. Improvements will be observed in constructs of the M-PAC framework.

## Methods:

### Study Design:

This study is an open-label, single-arm pre-post experimental trial with an optional 9-week follow up. The study design, procedures, and data analyses will largely follow those of previous non-randomized feasibility and efficacy studies [e.g., 23, 24, 25]. We adhered to the definitions of feasibility trials as outlined in the 2010 Consolidated Standards of Reporting Trials (CONSORT) guidelines [22, 26]. This non-randomized pre-post study with the primary aim of evaluating feasibility will inform future RCTs [27]. In addition, we will evaluate preliminary efficacy of the intervention and report estimations, particularly regarding behavioural outcome. The reporting of this protocol follows a guide proposed by Thabane and Lancaster [28], recommending the use of the SPIRIT (Standard Protocol Items: Recommendations for Interventional Trials) checklist [29], supplemented by items from the CONSORT 2010 statement (see Multimedia Appendix 1). The final study will follow a guide proposed by Lancaster and Thabane [30], recommending the use of the STROBE (Strengthening the Reporting of Observational Studies in Epidemiology) statement alongside with the CONSORT extension for pilot and feasibility trials. Ethical approval is obtained from Research Ethics Boards at the University of British Columbia (UBC) (H24-01820). Any substantive protocol modifications (e.g., amendments that can affect study validity) [29] will be formally reviewed and approved by the Research Ethics Boards. A list of amendments will be transparently reported in the final study. This trial is registered at ClinicalTrials.gov (NCT06573125).

### Participation eligibility:

This study will include outpatients aged 18–64 years who self-report a diagnosis of major depressive disorder or self-report at least mild depressive symptoms, as indicated by scoring at least 5 on the 9-item Patient Health Questionnaire (PHQ-9) [31]. Participants will be required to demonstrate literacy in English, possess an active email address, and own either an iPhone or an Android smartphone with internet capability to download the Pathverse app to access the MoodMover program. This study will include individuals who self-report engaging in less than 90 minutes of moderate-vigorous PA per week. Concomitant care and interventions will be allowed during the trial; participants may continue using medications provided the dosage remains unchanged. All participants must give fully informed consent on REDCap [32]. Exclusion criteria include self-reporting of a physical disability and/or health condition that prevents exercise (e.g., unstable angina, uncontrolled diabetes, acute heart failure), active psychosis or mania, active suicidal thoughts, severe cognitive impairment (e.g., major neurocognitive disorder), and/or being currently pregnant. In addition, individuals will not be eligible if they anticipate a major absence (e.g., vacation, surgery) in the next three months.

### Recruitment:

Patients will be recruited using multiple strategies. First, a brief introduction to this research study will be posted on REACH BC [33] and other research institute websites in Canada (e.g., [34]). Second, UBC mental health counseling services and other healthcare providers across Canada, as well as free depression advocacy groups (potential groups can be found at [35, 36]), will be approached for potential dissemination of study information. Third, posters will be placed in different hospitals and clinics in British Columbia, Canada. Notably, remote recruitment will be employed to mimic real-world conditions, and not all recruitment strategies will be implemented simultaneously. The selection and modification of recruitment strategies will consider available resources, time constraints, and the speed of recruitment. Potential participants will complete a screening questionnaire on REDCap (see Multimedia Appendix 2). Individuals who are found ineligible due to active suicidal thoughts will be informed immediately via email, and they will be provided with information about other community resources available for mental health support. Those who meet the eligibility criteria will be invited to participate in the study. Interested and eligible individuals will receive detailed study information via email and will complete the informed consent process electronically and all baseline measures of interest on REDCap. Potential participants will be given one week to consider the provided information, and they will receive a second invitation to confirm their participation through email. Participants can withdraw from the treatment or the study at any time.

### Procedures:

After an electronical consent form and baseline measures are completed, a researcher will register participants to the MoodMover program on the Pathverse admin web portal using their provided email address. Participants will receive an email with a MoodMover user guide, an instructional letter including information about downloading the Pathverse app from either the Apple Store or Google Play, and how to share their smartphone screens over Zoom. In particular, they will be instructed to sign up and log into Pathverse using the email address they provided.

Participants will be required to take a 15-minute one-on-one orientation session via Zoom over their smartphones. Before the orientation, participants will be asked to read through the MoodMover user guide. During the orientation, participants will share their smartphone screens and complete the first introduction module themselves while the researcher observes and provides explanations as needed, particularly regarding the ramped step goal recommendations. Following the completion of the first module, the researcher will hold a brief Q&A session to address any remaining questions or concerns the participant may have. Detailed scripts will be used to ensure consistent delivery of information across participants. Five orientation sessions will be recorded and assessed by a research assistant using a fidelity checklist after each session, which will allow immediate modifications to improve treatment fidelity [37]. The start date of the program will be set as the day of the orientation session. Participants can postpone the start date if needed by informing the researcher via email.

After completing the intervention, participants will undergo a re-assessment on REDCap. Participants will be encouraged to keep the app on their smartphones and continue using the app after completing the program. Choosing not to uninstall the app will be considered as providing consent, allowing the researcher to access and track their user engagement for a follow-up at 9 weeks. These participants will be approached on the last day of the follow-up period to sync their steps with MoodMover again, allowing the researcher to collect their PA data during the entire follow-up period. In addition, participants will be asked to complete a satisfaction questionnaire on REDCap. All study participants will be compensated $10 or gifts of equivalent value for completing each pre- and post-intervention assessment. The enrollment, intervention, and assessment schedule, following the recommended format by the SPIRIT guidelines, aligning with relevant items from the CONSORT 2010 guidelines [26], is presented in Table 1.

Table 1. The schedule of enrollment, interventions, and assessments

|  | Study period | | | | |
| --- | --- | --- | --- | --- | --- |
| Activity | Screening | Enrollment | Intervention | Close-out | Follow-up |
| Time point (in weeks) | −t_1_ | 0 | 1-9 | 10 | 18 |
| Enrollment |  |  |  |  |  |
| Screening questionnaires | ✔ |  |  |  |  |
| Informed consent |  | ✔ |  |  |  |
| Demographics and clinical information^a^ |  | ✔ |  |  |  |
| Intervention |  |  |  |  |  |
| MoodMover |  |  |  |  |  |
| Assessments |  |  |  |  |  |
| Primary feasibility and acceptability outcomes |  |  |  |  |  |
| Recruitment rate |  | ✔ |  |  |  |
| Retention rate |  |  |  | ✔ |  |
| User engagement |  |  |  | ✔ | ✔ |
| Patient-version MAUQ for standalone apps |  |  |  | ✔ |  |
| Reasons for discontinuation |  |  | ✔ |  |  |
| Reasons for non-adherence |  |  | ✔ | ✔ |  |
| Satisfaction |  |  |  | ✔ | ✔ |
| Adverse events monitoring |  |  | ✔ |  |  |
| Secondary outcomes |  |  |  |  |  |
| PAAQ (self-reported) |  | ✔ |  | ✔ |  |
| Physical activity (smartphone-generated step counts) |  |  | ✔ |  | ✔ |
| PHQ-9 |  | ✔ |  | ✔ |  |
| GAD-7 |  | ✔ |  | ✔ |  |
| WHODAS 2.0 |  | ✔ |  | ✔ |  |
| M-PAC constructs |  | ✔ |  | ✔ |  |
| PSQI |  | ✔ |  | ✔ |  |

Note. GAD-7: Generalized Anxiety Disorder 7; MAUQ: mHealth App Usability Questionnaire; M-PAC: Multi-Process Action Control; PAAQ: Physical Activity Adult Questionnaire; PHQ-9: Patient Health Questionnaire – 9 items; PSQI: Pittsburgh Sleep Quality Index; WHODAS 2.0: World Health Organization Disability Assessment Schedule 2.0

^a^Includes: sex, gender, race/ethnicity, age, height, weight, the highest level of education, employment status, income, disease duration, medications use, other ongoing treatments relevant to depression management, substance use, and current and past usage of physical activity apps or devices;

### Intervention:

MoodMover is a 9-week app-based intervention designed to increase PA among people with depression delivered through Pathverse [18]. The content was contextualized for people with depression based on an existing 10-week, web-based PA intervention for young adults guided by the M-PAC framework [23]. The current version of MoodMover consists of one run-in module for introduction and baseline self-monitoring, eight major modules, and eight complementary (optional) modules. After completing the first week, participants will be encouraged to set daily step goals by increasing an additional 1000 steps above their baseline PA level (week 1) every two weeks until reaching an increment of 3000 steps per day. Multimedia Appendix 3 depicts each major lesson's topics. A combination of different formats has been employed for delivering educational content, including videos, podcasts, and illustrated articles.

The app incorporated a wide range of behavioral change techniques (BCTs; e.g., self-monitoring and graded tasks) following the BCT taxonomy V1 [38] to implement the behavioral constructs of the M-PAC model. Apart from structured psychoeducational content, the app also offers diverse behavioral features, allowing participants to set step goals and action plans, monitor their progress, and engage in anonymous communication with other participants on pre-determined topics related to exercising. To improve engagement and promote PA, MoodMover also incorporated brief notifications and a gamification element where participants can earn 20 points for completing each major lesson and 10 points for completing each complementary lesson. Every 60 points earned is equivalent to a $5 CAD e-gift card. Screenshots in Figure 1 illustrate the home screen, step tracker, exercise log (with mood monitoring after exercising), and a card of a major lesson of the refined prototype of MoodMover. Screenshots in Figure 2 illustrate the community forum of MoodMover. The program details are described elsewhere (Tang et al. [18]). Participants will be instructed to read the lessons and complete all activities within each lesson over a 9-week period, with one lesson (week 1) and two lessons (one major and one complementary lesson; week 2-9) released at pre-scheduled times per week.
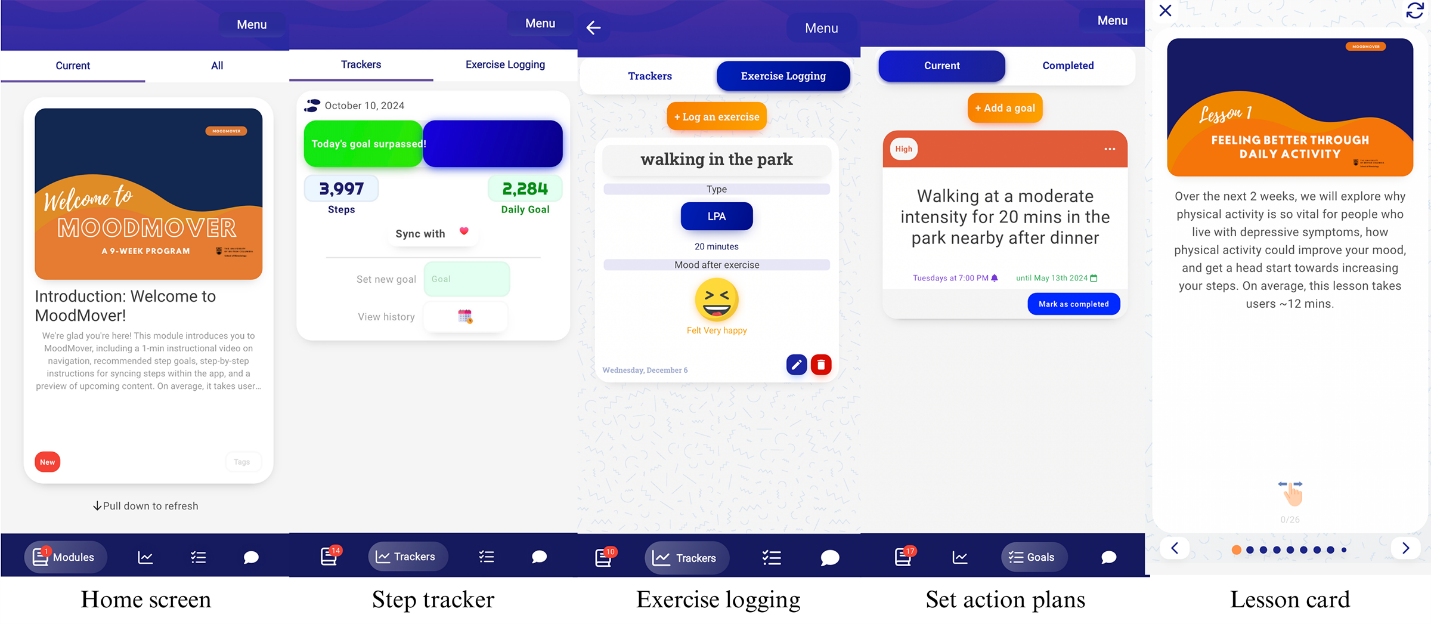


***Figure 1****. Screenshots of home screen, step tracker, action planning, exercise logging and a card of a major lesson*

*
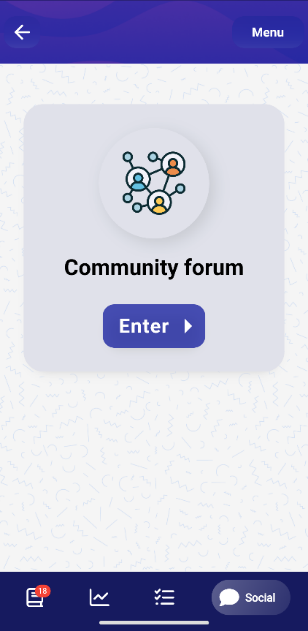

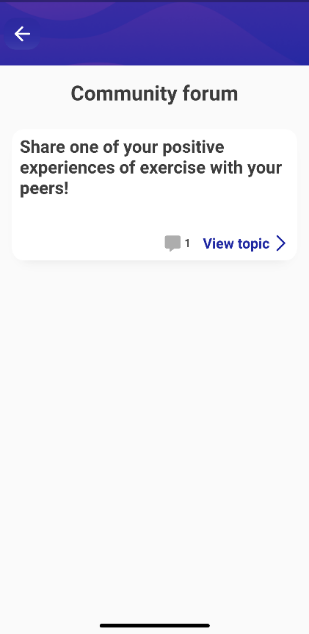

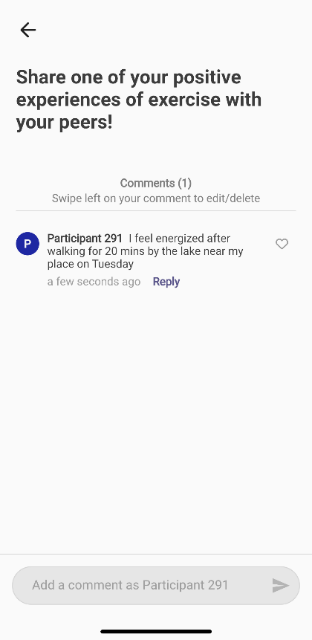
*

***Figure 2****. Screenshots of the peer-to-peer communication forum*

### Measures:

#### Demographic and clinical information:

All participants will be asked to provide demographic and clinical information (see Multimedia Appendix 4), such as sex and gender, race/ethnicity, age, height, weight, the highest level of education, employment status, income, duration of depression, medication use, other ongoing treatments relevant to depression management, substance use (e.g., cannabis), and current and past usage of physical activity apps or devices.

#### Primary feasibility and acceptability outcomes:

The traffic light system will be used to determine whether proceeding to a full-scale RCT based on the worst-performing feasibility criterion [39]. The 3-tiered progression criteria require setting cut-offs for green (proceed without changes), amber (proceed with amendments), and red (stop proceeding) zones for each of the key feasibility criterion. Table 2 presents the pre-determined thresholds for recruitment, adherence, usability, and retention based on previous literature, our usability testing study of MoodMover [18], pragmatic considerations, and expert insight within the research team. In addition, compliance to the graded goal setting and user satisfaction will be assessed to support the interpretation of feasibility and acceptability.

*Recruitment.* Recruitment rate will be calculated by dividing the number of people with depression who enrolled in the trial by the number of interested individuals meeting the eligibility criteria. Successful recruitment will be defined as achieving a rate of 65%. This progression criteria was based on studies with similar feasibility-based designs and those sampling from adults with mental illness [e.g., 40]. The time taken, source of recruitment, and the number of recruitment sites (if placed posters) to recruit the targeted sample size will also be recorded.

*Adherence.* Intervention adherence will be based on the proportion of participants who complete the majority (five out of eight) of major lessons. Previous systematic reviews identified a wide range of adherence rates for mHealth apps among people with depression. For example, 2/3 of studies in Serrano-Ripoll et al. [41] demonstrated a adherence rate of less than 50% (if reported). In contrast, Kerst et al. [42] observed adherence rates ranging from 70% to 94% in the majority (5/7) of the reviewed studies; however, remarkably lower adherence rates (22% and 35%) in two studies. In this study, an adherence rate of 70% will be deemed as falling within the green zone. Other user engagement metric data (i.e., time spent on each module) will be also downloaded from the Pathverse admin web portal. Various available objective engagement metrics, as summarized in Molloy and Anderson [43], will be calculated and reported, including use of specific program features (e.g., number of times participants changed their step goals, number of action plans completed, and number of exercise sessions logged), program use by the number of active days (i.e. number of days the program is used at least once), total duration of use, and average duration for completing the major and complementary modules. Participants who complete all 8 major modules will be categorized as “complete users”; those who complete 2-8 modules will be categorized as “incomplete users”; and those who do not use the app after the orientation session will be categorized as “nonusers”, respectively. Ongoing monitoring of adherence will be employed. Participants who have not opened the app for two weeks will be contacted for a check-in via email. Reasons for non-adherence (if provided) will be recorded.

*Usability.* The adapted, patient version of the mHealth app usability questionnaire (MAUQ) [44] designed for standalone apps will be employed. The MUAQ contains 13 items (see Multimedia Appendix 5 and Tang et al. [18]). It evaluates three domains of usability as defined by the International Organization for Standardization (ISO) definition of usability [45]: ease of use (MAUQ_E; 5 items, e.g., “The app was easy to use”), usefulness (MAUQ_U; 1 item, e.g., “The app would be useful for my mental health and well-being”), and interface and satisfaction (MAUQ_I; 7 items, e.g., “The information in the app was well organized, so I could easily find the information I needed.”). Participants will be asked to rate each item on a 7-point Likert scale (1 = strongly disagree to 7 = strongly agree). An average score of 5 and over will be deemed as acceptable consistent with Tang et al. [18].

*Retention rates.* Retention will be determined by the proportion of participants completing both pre- and post-intervention questionnaires. Torous et al. [46] found an average attrition rate of 26.2%, rising to 47.8% when adjusted for publication bias, among mental health apps for depression, with lower rates observed in those with mood tracking (18.4%) and human feedback (11.7%). An attrition rate of 30% will be defined as acceptable (green zone) for MoodMover. Additionally, reasons for discontinuation and any adverse events will be recorded.

*Compliance.* The compliance rate to the graded goal setting will be reported. Specifically, the proportion of participants who successfully set their step goal in week 2, and those who modified their step goals in week 4 and week 6, will be reported separately. Participants who followed all the instructions across these time points will be deemed as having achieved “full compliance”, while those who followed instructions at one or two time points will be deemed as having achieved “partial compliance”. Participants who did not follow instructions at any time points will be deemed as having achieved “no compliance”.

*Satisfaction.* Users’ satisfaction levels will be evaluated using Melin et al.’s mHealth Satisfaction Questionnaire [47] at both post-intervention and the 9-week follow-up. This 14-item questionnaire is recommended by Hajesmaeel et al. [48] as it was specifically designed for mHealth applications. Participants will be asked to rate on a 5-point Likert scale (1 = strongly disagree to 5 = strongly agree). Among 14 items, four items are negatively stated and will be reversed in the analyses. The total score ranges from 14 to 60, with a higher score indicating a higher satisfaction level. In addition, participants will be asked to list three things they liked and disliked about the intervention.

Table 2.  Pre-determined progression criteria.

| Feasibility outcomes | Green zone (go) | Amber zone (amend) | Red zone (stop) |
| --- | --- | --- | --- |
| **Recruitment** | ≥65% of eligible participants will be randomized | 40%- <65% of eligible participants will be randomized | <40% of eligible participants will be randomized |
| **Adherence** | ≥70% of participants complete the minimum requirements, that is, completing 5 major lessons | 40%- <70% of participants complete the minimum requirements, that is, completing 5 major lessons | <40% of participants complete the minimum requirements, that is, completing 5 major lessons |
|  |  |  |  |
| **Retention** | ≥70% participants complete the follow-up assessment | 40%-<70% participants complete the follow-up assessment | <40% participants complete the follow-up assessment |
| **Usability** | ≥70% of participants scored 5 and over on MAUQ | 40%- <70% of participants scored 5 and over on MAUQ | <40% of participants scored 5 and over on MAUQ |
|  |  |  |  |

*Note.* MAUQ: mHealth App Usability Questionnaire

#### Secondary outcomes:

While this study is not powered to determine the intervention's efficacy definitively, we will still collect data on each outcome to investigate interval estimates of the changes. The response rate and completion rate of each measure will be reported to inform the feasibility of administering the selected measures in a future trial. Additionally, we aim to assess the potential of MoodMover to produce clinically significant PA and depression changes in a larger evaluation.

*Physical activity (behavioral outcome).* Change in daily step counts will be collected using the smartphone’s built-in step counting function, which will be synced with MoodMover and can be downloaded from the Pathverse admin web portal. A significant increase of 3000 daily steps for 5 days per week will be deemed as reaching the clinically significant behavioral goal of MoodMover [18]. Moreover, to evaluate self-reported PA, we will utilize the Canadian Physical Activity Adult Questionnaire (PAAQ) [49]. This questionnaire is designed in accordance with the Canadian Physical Activity Guidelines, which recommend a minimum of 150 minutes of MVPA per week for adults (see Multimedia Appendix 6). It has been widely employed for monitoring PA in Canadian adults and has demonstrated a stronger association with accelerometer-based data than the International Physical Activity Questionnaire [49, 50]. Along with the PAAQ questionnaire, participants will be asked at post-intervention whether they have linked a smartwatch or fitness device to Health (iOS) or Google Fit (Android) throughout the program. Additionally, they will be asked how often (almost always, sometimes, seldom) they carried their phone (or wore their smartwatch, if used) during their non-sedentary waking hours during workdays and weekend (“How frequently do you carry your phone/wear your smartwatch with you during your non-sedentary waking hours?”).

*Depressive symptoms (clinical outcome).* Depression will be evaluated utilizing the PHQ-9 [31], a widely used self-report scale that demonstrates sensitivity in detecting changes after treatment in psychiatric patients [51]. Participants will be asked to rate 9 items, using 0-to-3 Likert scales, resulting in a total score ranging from 0 to 27 (see Multimedia Appendix 7). Higher scores reflect more severe depression. The classification of depression severity will be as follows: a score of 5-9 will be considered indicative of mild depression, 10-19 as moderate, and 20-27 as severe [31]. To define a clinically meaningful change in depression, McMillan et al. [52] proposed a reduction of 5 points on the PHQ-9, along with a PHQ-9 score shift from ≥ 10 at baseline to ≤ 9 at post-intervention to be considered clinically relevant. Given that individuals with mild depression will be eligible for the current study, this classification will be exclusively applied to those who scored 10 or higher at baseline.

*Anxiety*. Anxiety will be measured by the Generalized Anxiety Disorder 7 scale (GAD-7) [53] (See Multimedia Appendix 8). The GAD-7 is a seven-item validated [54] instrument for measuring the severity of anxiety on four-point Likert scales (0 = “not at all” to 3 = “nearly every day”). The total score on the scale ranges from 0 to 21, with higher scores indicating severe anxiety. The classification of anxiety severity will be as follows: 5 for mild, 10 for moderate and 15 for severe.

*Sleep quality*. The Pittsburgh Sleep Quality Index (PSQI) [55] is a self-rated, 19-item questionnaire with strong reliability and validity [56] that assesses various sleep-related problems over the past month (see Multimedia Appendix 9). The 19 questions are categorized into seven components, including sleep quality, sleep duration, sleep latency, habitual sleep efficiency, sleep disorders, use of sleep medication, and daytime dysfunction. Participants will be asked to rate on a 4-point Likert scale ranging from 0 to 3. A total score ranging from 0 to 21 can be summed for these seven components, with a higher score reflecting poorer sleep quality. A total score of >5 indicates a poor quality of sleep.

*Functional disability.* World Health Organization Disability Assessment Schedule 2.0 (WHODAS 2.0) [57] will be utilized to evaluate participants' health and ability to perform activities in six areas of functionality: cognition, mobility, self-care, getting along, life activities, and participation (See Multimedia Appendix 10). The WHODAS 2.0 comprises 12 items. For each item, participants will be asked to estimate the extent of their disability over the past 30 days, using a 5-point Likert scale ranging from 1 (none) to 5 (extreme/cannot do). The total score on the scale ranges from 0 to 100, with higher scores indicating a greater degree of disability. WHODAS 2.0 has good internal consistency across all domains (α = .77 - .98) [58].

M-PAC constructs:

The measures of the reflective and regulatory processes within the M-PAC framework will be primarily derived from Tang et al. [59]. The M-PAC survey is presented in Multimedia Appendix 11.

*Reflective processes. Affective attitudes* and *instrumental attitudes* will be measured on three 7-point bipolar scales, respectively. All items share the same stem: “For me, participating in regular PA over the next month would be ...” Higher scores indicate more positive attitudes. The items had demonstrated very good to excellent inter-item reliability in Tang et al. [59] (affective attitudes, α = .87; instrumental attitudes, α = .91). *Perceived opportunity* and *perceived capability* will be assessed using three 5-point Likert scales, ranging from 1 (strongly disagree) to 5 (strongly agree), respectively. The items have shown acceptable inter-item reliability (opportunity, α = .70; capability, α = .67) [60].

*Intention strength* will be measured using three items (e.g., I am committed to engage in physical activity over the next month). Participants will rate these items on 5-point Likert scales ranging from 1 (strongly disagree) to 5 (strongly agree). The items have exhibited very good inter-item reliability (α = .89). In addition, *decisional intention* will be evaluated using one item regarding the intention to perform more PA and achieve the behavioral outcome goal of increasing 3000 daily steps above their baseline PA levels for most days per week. Participants will be asked to provide binary responses (Yes/No).

*Regulatory processes. Regulations* will be measured using 5-point Likert scales with three items pertaining to self-monitoring, goal-setting, and action planning. The inter-item reliability for the regulatory process measures has been found to be very good (α = .81).

*Reflexive processes. Habit* will be assessed using three items (e.g., "I engage in regular physical activity without having to consciously remember it"), which have been modified from Rhodes and Lim [61] and were originally derived from the subscale of the Self-Report Habit Index [62]. *Identity* will be measured using three items (e.g., "When I describe myself to others, I usually include my involvement in physical activity"), which have been adapted from previous studies [61, 63]. Both measures will utilize a 5-point Likert scale ranging from 1 (strongly disagree) to 5 (strongly agree) for participant responses. The inter-item reliability of the habit measure has demonstrated excellent internal consistency (α = .93), while the identity measure has shown very good internal consistency (α = .87) [61].

### Sample size justification:

As a single-arm feasibility trial, no formal sample size calculation was performed. Instead, the sample size was informed by the recommendations for the intervention arm of pilot RCTs, based on the "traffic light" progression criteria [39]. Four feasibility criteria (i.e., recruitment, adherence, usability, and retention) were considered individually (see Table 2). To achieve over 90% statistical power for each criterion, as aligned with the quick look-up grid for sample size based on normal approximation approach in Lewis et al. [39], the study requires: (1) 36 participants for recruitment; (2) 25 participants in the intervention group to assess adherence; (3) 25 participants in the intervention group to assess usability; and (4) 25 participants for retention. The overall sample size was determined by the criterion with the highest requirement—adherence and usability, requiring 25 participants per arm. Considering a 30% attrition rate, the sample size for adherence increases to 36 participants. With an expected 65% recruitment success as our previous usability testing study indicated, 55 eligible patients need be invited to achieve the target sample size (55 = 1/0.65 × 36). Considering the available resources and time, a sample size of 20 for the intervention arm, as suggested by Lancaster et al. [64] for pilot studies, will also be considered acceptable.

### Data management

During data collection, all questionnaire data will be collected and collated on the UBC-hosted data management platform, REDCap. App-related data will be collected by Pathverse and stored in Canada on Amazon Web Services (AWS) [65]. Five orientation sessions will be transcribed using Otter.ai [66], which stores data in the AWS West region, United States. After the intervention, all data will be downloaded and securely stored on a UBC-secured, password-protected computer, and then transferred to UBC's secure OneDrive. All files will be password-protected and encrypted. Only authorized researchers involved in the study will have access to the data. After data collection is completed, any personal identifying information will be deleted. As is common practice in scientific research, other researchers may request access to the de-identified data after publications. Participants will be fully informed of these procedures before consenting to this trial.

### Statistical analysis

All analyses will be conducted using either SPSS ver. 29 or R Studio. Descriptive statistics will be presented for all measures. The strategy for addressing missing data will be determined by evaluating the degree and pattern of missingness. The impact of influential factors (such as baseline characteristics, including depression severity) on feasibility results, in particular engagement, will be explored using a regression-based analysis. Additionally, we will explore the preliminary impact of MoodMover on secondary outcomes (e.g., PA, depression, and M-PAC constructs) and investigate using random effects mixed modelling. As an underpowered single-arm feasibility study, the reporting of efficacy outcomes will focus on estimation. Results will be reported with 75%, 85%, and 95% confidence intervals, while considering clinically meaningful changes as suggested by Lee et al [67] and Walters [68]. A binary variable will be generated to indicate whether a participant met the behavioral step goal (1) or not (0). Specifically, the pre-determined clinically meaningful decrease in depression will be incorporated into the interpretation of the results for those who scored 10 or above on the PHQ-9 at baseline. A *p*-value < 0.05 will be considered statistically significant.

### Harms and data monitoring

The research protocol described here is categorized with low research risk and low to moderate participant vulnerability. The intervention is not anticipated to cause physical harm. The app focuses on promoting lifestyle physical activities, which carry no greater risk than participants would experience in their daily lives. Although using an app for data collection introduces moderate privacy and confidentiality risks, the Pathverse platform employs strong technical standards and encryption. Access to the Pathverse web portal is password-protected, and all collected data is deidentified and encrypted during transit and storage. Therefore, privacy and confidentiality risks are considered low. Participants, who may have varying levels of depression or other mental health issues, represent a vulnerable group. They might experience a change in their mental health status inherently or emotional distress if unable to meet daily step goals. As a remote study, researchers will not be able to provide in-person and/or immediate support for participants, placing this risk in the moderate range. However, concomitant care is permitted, including stable medication use, contributing to a low to moderate level of participant vulnerability. Additionally, participants will be provided with information on 24/7 mental health supports (e.g., national crisis lines) in case of urgent need. As this trial has a short duration (9 weeks) and generally low-risk considerations, a data monitoring committee is not incorporated.

## Results

Recruitment is anticipated to begin by October 2024. Data collection and analysis are expected to be completed by June 2025. Trial results will be disseminated via publications in peer-reviewed journals and via presentations at academic conferences.

## Discussion

To provide greater evidence-based treatment options for patients with depression who are not interested in, or have no access to conventional depression treatments, well-designed app-based intervention that can increase PA and sustain those increases over time may be beneficial. This trial will explore the feasibility, usability, and acceptability of a 9-week, theory-based (M-PAC) app designed for people with depression following a combination of two systematic and rigorous behavioral intervention development frameworks (IDEAS and ORBIT). This approach is crucial for refining the intervention, improving study procedures, and informing the design of future definitive studies [26]. In addition, we will explore the potential effect of MoodMover on participants’ behavioral (PA) and clinical outcomes (e.g., depression). We hypothesize that this intervention will demonstrate potential in increasing multiple M-PAC constructs, such as affective attitudes, regulatory skills, habit, and identity. Both habit and identity are powerful theoretical constructs that we hope can produce sustained, long-term change in exercise behavior patterns.

One strength of this study is that the current version of MoodMover has passed usability testing among the target population and has been refined based on participant feedback [18]. In addition, MoodMover was designed based on the M-PAC framework, a behaviour change framework that has been deemed suitable for people with poor mental health [59], and incorporated a series of BCTs corresponding to the M-PAC constructs, along with additional BCTs to enhance engagement. Moreover, this study will include a more robust analysis of user engagement data powered by the Pathverse platform, whereas most previous studies with mHealth apps for depression reported only one or two metrics [43]. Researchers can closely track PA behavior (i.e., daily step counts) and the timing and reasons for app discontinuation, shedding light on the app usage lifecycle. In addition, an extensive range of potential influential factors will be assessed, aiding in the understanding of the associations between engagement and other variables. Understanding how app use changes over extended periods of time, and assessing various mediating variables and process measures, will provide insights into the mechanisms behind the interventions' success or failure and inform either moving to a larger efficacy trial, moving backwards to the previous phases of the IDEAS model for refinement, or abandoning further development of MoodMover.

The major limitation is the common high dropout rates and low user engagement often faced by mHealth apps for depression [46] could further impact the evaluation of MoodMover's efficacy. Torous et al. [46] found that apps incorporating mood monitoring showed a considerably lower dropout rate of 18.4%. Given this, we have incorporated a mood monitoring element by asking participants to rate their mood after exercising when they log their exercise behaviors. Moreover, to enhance the likelihood of sustained app usage, we have introduced several evidence-based elements: a gamification feature with incentives, a peer-to-peer support forum, reminders/notifications, an easy-to-use interface, and a combination of multiple formats to deliver educational content (see Tang et al. [18]). We anticipate that these strategies will increase adherence and engagement while potentially improving MoodMover's efficacy in promoting PA.

Overall, this study fits within Phase IIa and Phase IIb of the ORBIT model. The present study will provide insights into the potential of MoodMover serving as an alternative strategy or adjunct treatment for managing mild-to-moderate depression, which can be implemented into clinical mental health care in Canada. Despite the possibility of null efficacy results, this study will yield valuable data related to recruitment plans, trial processes and procedures (e.g., core assessments and app features), resources, trial management, and other feasibility variables (e.g., user engagement and satisfaction). In contrast, promising results will allow us to move to the next step, Phase IIc: Phase II Efficacy Trial, of the ORBIT model for testing the efficacy of MoodMover on behavioural outcome in an appropriately powered RCT. Other study designs, such as sequential, multiple assignment, randomized trials (SMARTs) [69], Just-in-Time Adaptive Interventions (JITAIs) [70], micro-randomized trials [71], single-case designs [72], dose-finding methods, may also be considered for assessing MoodMover in the future.

## Acknowledgements

YT and GF conceived the original study idea. YT drafted the manuscript. MG, HL, SL, RL, and GF reviewed the manuscript. HL provided technical support in the development of the app. All authors approved the final manuscript. This work was supported by a 4-Year Doctoral Fellowship from the University of British Columbia to YT, a Michael Smith Health Research BC Research Trainee Award to MG, Michael Smith Health Research BC Research Scholar Award to SL, and a Canadian Institute of Health Research (CIHR) Foundation Award to GF.

## Conflicts of Interest

The co-authors, SL and HL, are co-founders of Pathverse Inc.

## References

1. World Health Organization. Depressive disorder (depression). 2023 [cited 2024 May 21]; Available from: <https://www.who.int/news-room/fact-sheets/detail/depression>.

2. World Health Organization. Depression. 2021 [updated 13 September 2021; cited 2023 October 4th]; Available from: <https://www.who.int/news-room/fact-sheets/detail/depression>.

3. Statistics Canada. Mental health care needs, 2018. 2019 [cited 2023 Sep. 20th]; Available from: <https://www150.statcan.gc.ca/n1/pub/82-625-x/2019001/article/00011-eng.htm>.

4. Araya R, Zitko P, Markkula N, Rai D, Jones K. Determinants of access to health care for depression in 49 countries: a multilevel analysis. Journal of Affective Disorders. 2018;234:80-8. PMID: 29524750. doi: 10.1016/j.jad.2018.02.092.

5. Anokye NK, Trueman P, Green C, Pavey TG, Taylor RS. Physical activity and health related quality of life. BMC public health. 2012 Aug 7;12(1):1-8. PMID: 22871153. doi: 10.1186/1471-2458-12-624.

6. Kraus WE, Powell KE, Haskell WL, Janz KF, Campbell WW, Jakicic JM, et al. Physical activity, all-cause and cardiovascular mortality, and cardiovascular disease. Medicine and science in sports and exercise. 2019 Jun;51(6):1270. PMID: 31095084. doi: 10.1249/MSS.0000000000001939.

7. Centers for Disease Control and Prevention. Benefits of Physical Activity. n.d. [updated June 16th, 2022; cited 2024 May 24]; Available from: <https://www.cdc.gov/physicalactivity/basics/pa-health/index.htm#:~:text=Being%20physically%20active%20can%20improve,activity%20gain%20some%20health%20benefits>.

8. Heissel A, Heinen D, Brokmeier LL, Skarabis N, Kangas M, Vancampfort D, et al. Exercise as medicine for depressive symptoms? A systematic review and meta-analysis with meta-regression. British journal of sports medicine. 2023. PMID: 36731907. doi: 10.1136/bjsports-2022-106282.

9. Ravindran AV, Balneaves LG, Faulkner G, Ortiz A, McIntosh D, Morehouse RL, et al. Canadian Network for Mood and Anxiety Treatments (CANMAT) 2016 clinical guidelines for the management of adults with major depressive disorder: section 5. Complementary and alternative medicine treatments. The Canadian Journal of Psychiatry. 2016 Sep;61(9):576-87. PMID: 27486153. doi: 10.1177/0706743716660290.

10. Cooney GM, Dwan K, Greig CA, Lawlor DA, Rimer J, Waugh FR, et al. Exercise for depression. Cochrane database of systematic reviews. 2013 (9). PMID: 24026850. doi: 10.1002/14651858.CD004366.pub6.

11. McKay FH, Cheng C, Wright A, Shill J, Stephens H, Uccellini M. Evaluating mobile phone applications for health behaviour change: A systematic review. Journal of telemedicine and telecare. 2018;24(1):22-30. PMID: 27760883. doi: 10.1177/1357633X16673538.

12. Fitzgerald M, McClelland T. What makes a mobile app successful in supporting health behaviour change? Health education journal. 2017;76(3):373-81. doi: <https://doi.org/10.1177/0017896916681179>.

13. Middelweerd A, Mollee JS, van der Wal CN, Brug J, Te Velde SJ. Apps to promote physical activity among adults: a review and content analysis. International journal of behavioral nutrition and physical activity. 2014;11(1):1-9. PMID: 25059981. doi: 10.1186/s12966-014-0097-9.

14. Arigo D, Jake-Schoffman DE, Wolin K, Beckjord E, Hekler EB, Pagoto SL. The history and future of digital health in the field of behavioral medicine. J Behav Med. 2019;42:67-83. PMID: 30825090. doi: 10.1007/s10865-018-9966-z.

15. Direito A, Pfaeffli Dale L, Shields E, Dobson R, Whittaker R, Maddison R. Do physical activity and dietary smartphone applications incorporate evidence-based behaviour change techniques? BMC Public Health. 2014;14:1-7. PMID: 24965805. doi: 10.1186/1471-2458-14-646.

16. Bondaronek P, Alkhaldi G, Slee A, Hamilton FL, Murray E. Quality of publicly available physical activity apps: review and content analysis. JMIR mHealth and uHealth. 2018;6(3):e9069.

17. Tang Y, Gierc M, Lam RW, Liu S, Faulkner G. The effectiveness of internet-guided self-help interventions to promote physical activity among individuals with depression: systematic review. JMIR Mental Health. 2022;9(12):e38049. PMID: 36508243. doi: 10.2196/38049.

18. Tang Y, Gierc M, La. H, Kim J, Liu S, Lam RW, et al. MoodMover: Development and usability testing of an mHealth physical activity intervention for depression2024 Aug 9 2024 [cited 2024 Aug 9].

19. Rhodes RE. The evolving understanding of physical activity behavior: a multi-process action control approach. Advances in motivation science: Elsevier; 2017. p. 171-205.

20. Mummah SA, Robinson TN, King AC, Gardner CD, Sutton S. IDEAS (Integrate, Design, Assess, and Share): a framework and toolkit of strategies for the development of more effective digital interventions to change health behavior. Journal of medical Internet research. 2016;18(12):e5927. PMID: 27986647. doi: 10.2196/jmir.5927.

21. Liu S, La H, Willms A, Rhodes RE. A “No-Code” App Design Platform for Mobile Health Research: Development and Usability Study. JMIR Form Res. 2022 2022/8/18;6(8):e38737. doi: 10.2196/38737.

22. Powell L, Freedland KE, Kaufmann PG. Behavioral clinical trials for chronic diseases: Springer; 2020. ISBN: 978-3-030-39330-4.

23. Ross A. A feasibility study evaluating an online physical activity intervention for young adults with low mood and/or depression engaged in community primary clinical care: University of Victoria; 2023.

24. Takahashi K, Takada K, Hirao K. Feasibility and preliminary efficacy of a smartphone application intervention for subthreshold depression. Early Interv Psychiatry. 2019 Feb;13(1):133-6. PMID: 29356332. doi: 10.1111/eip.12540.

25. Inch J, Notman F, Bond CM, Alldred DP, Arthur A, Blyth A, et al. The Care Home Independent Prescribing Pharmacist Study (CHIPPS)-a non-randomised feasibility study of independent pharmacist prescribing in care homes. Pilot Feasibility Stud. 2019;5:89. PMID: 31338204. doi: 10.1186/s40814-019-0465-y.

26. Eldridge SM, Chan CL, Campbell MJ, Bond CM, Hopewell S, Thabane L, et al. CONSORT 2010 statement: extension to randomised pilot and feasibility trials. Pilot and Feasibility Studies. 2016 2016/10/21;2(1):64. doi: 10.1186/s40814-016-0105-8.

27. Bond C, Lancaster GA, Campbell M, Chan C, Eddy S, Hopewell S, et al. Pilot and feasibility studies: extending the conceptual framework. Pilot Feasibility Stud. 2023 Feb 9;9(1):24. PMID: 36759879. doi: 10.1186/s40814-023-01233-1.

28. Thabane L, Lancaster G. A guide to the reporting of protocols of pilot and feasibility trials. Springer; 2019. p. 1-3.

29. Chan A-W, Tetzlaff JM, Gøtzsche PC, Altman DG, Mann H, Berlin JA, et al. SPIRIT 2013 explanation and elaboration: guidance for protocols of clinical trials. Bmj. 2013;346. PMID: 23303884. doi: 10.1136/bmj.e7586.

30. Lancaster GA, Thabane L. Guidelines for reporting non-randomised pilot and feasibility studies. Pilot Feasibility Stud. 2019;5:114. PMID: 31608150. doi: 10.1186/s40814-019-0499-1.

31. Kroenke K, Spitzer RL, Williams JB. The PHQ‐9: validity of a brief depression severity measure. Journal of general internal medicine. 2001;16(9):606-13. PMID: 11556941. doi: 10.1046/j.1525-1497.2001.016009606.x.

32. Harris PA, Taylor R, Thielke R, Payne J, Gonzalez N, Conde JG. Research electronic data capture (REDCap)—a metadata-driven methodology and workflow process for providing translational research informatics support. Journal of biomedical informatics. 2009;42(2):377-81.

33. REACH BC. [cited 2024 June 24]; Available from: <https://reachbc.ca/>.

34. Vancouver Coastal Health Research Institute. [cited 2024 June 24]; Available from: <https://www.vchri.ca/>.

35. Registered Nurses' Association of Ontario. [cited 2024 June 24]; Available from: <https://mharesource.rnao.ca/advocacy-groups-mental-health-canada>.

36. Mood Disorders Association of British Columbia. [cited 2024 June 24]; Available from: <https://mdabc.net/resources/mdabc-support-groups/>.

37. Teresi JA, Yu X, Stewart AL, Hays RD. Guidelines for designing and evaluating feasibility pilot studies. Medical care. 2022;60(1):95-103. PMID: 34812790. doi: 10.1097/MLR.0000000000001664.

38. Michie S, Richardson M, Johnston M, Abraham C, Francis J, Hardeman W, et al. The behavior change technique taxonomy (v1) of 93 hierarchically clustered techniques: building an international consensus for the reporting of behavior change interventions. Annals of Behavioral Medicine. 2013 Aug;46(1):81-95. PMID: 23512568. doi: 10.1007/s12160-013-9486-6.

39. Lewis M, Bromley K, Sutton C, McCray G, Myers H, Lancaster G. Determining sample size for progression criteria for pragmatic pilot RCTs: the hypothesis test strikes back! Pilot and Feasibility Studies. 2021;7(1):1-14. PMID: 33536076. doi: 10.1186/s40814-021-00770-x.

40. Firth J, Rosenbaum S, Stubbs B, Gorczynski P, Yung AR, Vancampfort D. Motivating factors and barriers towards exercise in severe mental illness: a systematic review and meta-analysis. Psychological medicine. 2016;46(14):2869-81. doi: <https://doi.org/10.1017/S0033291716001732>.

41. Serrano-Ripoll MJ, Zamanillo-Campos R, Fiol-DeRoque MA, Castro A, Ricci-Cabello I. Impact of smartphone app–based psychological interventions for reducing depressive symptoms in people with depression: Systematic literature review and meta-analysis of randomized controlled trials. JMIR mHealth and uHealth. 2022;10(1):e29621. PMID: 35084346. doi: 10.2196/29621.

42. Kerst A, Zielasek J, Gaebel W. Smartphone applications for depression: a systematic literature review and a survey of health care professionals’ attitudes towards their use in clinical practice. European archives of psychiatry and clinical neuroscience. 2020;270(2):139-52. PMID: 30607530. doi: 10.1007/s00406-018-0974-3.

43. Molloy A, Anderson PL. Engagement with mobile health interventions for depression: A systematic review. Internet Interventions. 2021;26:100454. PMID: 34621626. doi: 10.1016/j.invent.2021.100454.

44. Zhou L, Bao J, Setiawan IMA, Saptono A, Parmanto B. The mHealth App Usability Questionnaire (MAUQ): development and validation study. JMIR mHealth and uHealth. 2019;7(4):e11500. PMID: 30973342. doi: 10.2196/11500.

45. International Organization for Standardization. Ergonomics of human-system interaction — Part 11: Usability: Definitions and concepts. 2018 [cited 2024 May 27]; Available from: <https://www.iso.org/obp/ui/#iso:std:iso:9241:-11:ed-2:v1:en>.

46. Torous J, Lipschitz J, Ng M, Firth J. Dropout rates in clinical trials of smartphone apps for depressive symptoms: a systematic review and meta-analysis. Journal of Affective Disorders. 2020;263:413-9. PMID: 31969272. doi: 10.1016/j.jad.2019.11.167.

47. Melin J, Bonn SE, Pendrill L, Trolle Lagerros Y. A Questionnaire for Assessing User Satisfaction With Mobile Health Apps: Development Using Rasch Measurement Theory. JMIR Mhealth Uhealth. 2020 May 26;8(5):e15909. PMID: 32452817. doi: 10.2196/15909.

48. Hajesmaeel-Gohari S, Khordastan F, Fatehi F, Samzadeh H, Bahaadinbeigy K. The most used questionnaires for evaluating satisfaction, usability, acceptance, and quality outcomes of mobile health. BMC Med Inform Decis Mak. 2022 Jan 27;22(1):22. PMID: 35081953. doi: 10.1186/s12911-022-01764-2.

49. Colley RC, Butler G, Garriguet D, Prince SA, Roberts KC. Comparison of self-reported and accelerometer-measured physical activity in Canadian adults. Health Rep. 2018;29(12):3-15. PMID: 30566204.

50. Garriguet D, Tremblay S, Colley RC. Comparison of Physical Activity Adult Questionnaire results with accelerometer data. Health Reports. 2015;26(7):11. PMID: 26177042.

51. Beard C, Hsu K, Rifkin L, Busch A, Björgvinsson T. Validation of the PHQ-9 in a psychiatric sample. Journal of Affective Disorders. 2016;193:267-73. PMID: 26774513. doi: 10.1016/j.jad.2015.12.075.

52. McMillan D, Gilbody S, Richards D. Defining successful treatment outcome in depression using the PHQ-9: a comparison of methods. Journal of Affective Disorders. 2010;127(1-3):122-9. PMID: 20569992. doi: 10.1016/j.jad.2010.04.030.

53. Spitzer RL, Kroenke K, Williams JB, Löwe B. A brief measure for assessing generalized anxiety disorder: the GAD-7. Archives of internal medicine. 2006;166(10):1092-7. PMID: 16717171. doi: 10.1001/archinte.166.10.1092.

54. Löwe B, Decker O, Müller S, Brähler E, Schellberg D, Herzog W, et al. Validation and standardization of the Generalized Anxiety Disorder Screener (GAD-7) in the general population. Medical care. 2008:266-74. PMID: 18388841. doi: 10.1097/MLR.0b013e318160d093.

55. Buysse DJ, Reynolds III CF, Monk TH, Berman SR, Kupfer DJ. The Pittsburgh Sleep Quality Index: a new instrument for psychiatric practice and research. Psychiatry Research. 1989;28(2):193-213. PMID: 2748771. doi: 10.1016/0165-1781(89)90047-4.

56. Mollayeva T, Thurairajah P, Burton K, Mollayeva S, Shapiro CM, Colantonio A. The Pittsburgh sleep quality index as a screening tool for sleep dysfunction in clinical and non-clinical samples: A systematic review and meta-analysis. Sleep medicine reviews. 2016;25:52-73. PMID: 26163057. doi: 10.1016/j.smrv.2015.01.009.

57. Üstün TB. Measuring health and disability: Manual for WHO disability assessment schedule WHODAS 2.0: World Health Organization; 2010. ISBN: 9241547596.

58. Garin O, Ayuso-Mateos JL, Almansa J, Nieto M, Chatterji S, Vilagut G, et al. Validation of the" World Health Organization Disability Assessment Schedule, WHODAS-2" in patients with chronic diseases. Health and quality of life outcomes. 2010;8:1-15.

59. Tang Y, Gierc M, Whiteford V, Rhodes RE, Faulkner G. Exploring correlates of physical activity using the multi-process action control framework: is there a moderating role for mental health? International Journal of Sport and Exercise Psychology. 2023:1-19. doi: <https://doi.org/10.1080/1612197X.2023.2225515>.

60. Ursachi G, Horodnic IA, Zait A. How reliable are measurement scales? External factors with indirect influence on reliability estimators. Procedia Economics and Finance. 2015;20:679-86. doi: <https://doi.org/10.1016/S2212-5671(15)00123-9>.

61. Rhodes RE, Lim C. Understanding action control of daily walking behavior among dog owners: a community survey. BMC Public Health. 2016;16(1):1-9. PMID: 27852251. doi: 10.1186/s12889-016-3814-2.

62. Gardner B, Abraham C, Lally P, de Bruijn G-J. Towards parsimony in habit measurement: Testing the convergent and predictive validity of an automaticity subscale of the Self-Report Habit Index. International Journal of Behavioral Nutrition and Physical Activity. 2012;9(1):1-12. PMID: 22935297. doi: 10.1186/1479-5868-9-102.

63. Wilson PM, Muon S. Psychometric properties of the exercise identity scale in a university sample. International Journal of Sport and Exercise Psychology. 2008;6(2):115-31. doi: <https://doi.org/10.1080/1612197X.2008.9671857>.

64. Lancaster GA, Dodd S, Williamson PR. Design and analysis of pilot studies: recommendations for good practice. Journal of evaluation in clinical practice. 2004;10(2):307-12. PMID: 15189396. doi: 10.1111/j..2002.384.doc.x.

65. Amazon Web Services. [Aug 6]; 2024]. Available from: <https://aws.amazon.com/>.

66. Otter.ai. 2024; Available from: <https://otter.ai/>.

67. Lee EC, Whitehead AL, Jacques RM, Julious SA. The statistical interpretation of pilot trials: should significance thresholds be reconsidered? BMC Medical Research Methodology. 2014;14:1-8. PMID: 24650044. doi: 10.1186/1471-2288-14-41.

68. Walters SJ. Consultants' forum: should post hoc sample size calculations be done? Pharmaceutical Statistics: The Journal of Applied Statistics in the Pharmaceutical Industry. 2009;8(2):163-9. PMID: 18416448. doi: 10.1002/pst.334.

69. Lei H, Nahum-Shani I, Lynch K, Oslin D, Murphy SA. A “SMART” design for building individualized treatment sequences. Annual Review of Clinical Psychology. 2012;8. PMID: 22224838. doi: 10.1146/annurev-clinpsy-032511-143152.

70. Nahum-Shani I, Smith SN, Spring BJ, Collins LM, Witkiewitz K, Tewari A, et al. Just-in-time adaptive interventions (JITAIs) in mobile health: key components and design principles for ongoing health behavior support. Annals of Behavioral Medicine. 2018;52(6):446-62. PMID: 27663578. doi: 10.1007/s12160-016-9830-8.

71. Klasnja P, Hekler EB, Shiffman S, Boruvka A, Almirall D, Tewari A, et al. Microrandomized trials: An experimental design for developing just-in-time adaptive interventions. Health Psychol. 2015;34(S):1220. PMID: 26651463. doi: 10.1037/hea0000305.

72. Dallery J, Raiff BR. Optimizing behavioral health interventions with single-case designs: from development to dissemination. Translational behavioral medicine. 2014;4(3):290-303. PMID: 25264468. doi: 10.1007/s13142-014-0258-z.
