# Supplementary material for: Feasibility and preliminary effects of an app-based physical activity intervention for individuals with depression (MoodMover): A protocol for a single-arm, pre-post intervention study
Source: PLoS One. 2025 Apr 22;20(4):e0321958. doi: 10.1371/journal.pone.0321958 (PMC12013873; doi:10.1371/journal.pone.0321958)
Supplement: S3 File — (DOCX) [file pone.0321958.s003.docx]

**S3 File. Screening questionnaire.**

Chapter 1.

1. Are you aged between 18-64 years?

- Yes
- No (if selected, proceed to End of survey)

2. Do you have a smartphone, either iPhone or Android, with internet access that can download and use an app?

- Yes
- No (if selected, proceed to End of survey)

3. Are you able to read English? Are you able to respond to questions in spoken English?

- Yes
- No (if selected, proceed to End of survey)

4. Do you currently have a clinical diagnosis of a major depressive disorder?

- Yes
- No

5. If any, do you anticipate any major changes to your current antidepressant treatment regimen (e.g., changes in medication type or dosage, adjustments to psychotherapy) within the next 3 months?

- Yes (if selected, proceed to End of survey)
- No
- I’m not taking any antidepressant treatments

6. Do you currently have any physical disabilities that prevent you from engaging in regular exercise or physical activity?

- Yes (if selected, proceed to End of survey)
- No

7. Have you experienced any of the following conditions in the past month: active psychosis or mania, active suicidal thoughts, or severe difficulties with thinking and memory?

- Yes (if selected, proceed to End of survey)
- No

8. Do you have an active alcohol or substance use disorder?

- Yes (if selected, proceed to End of survey)
- No

9. If you are female, are you currently pregnant?

- Yes (if selected, proceed to End of survey)
- No
- Not applicable

10. Have you been diagnosed with another major primary psychiatric disorder, such as psychosis?

- Yes (if selected, proceed to End of survey)
- No

10. In the past week, on how many days have you done a total of 30 minutes or more of physical activity, which was enough to raise your breathing rate? This may include sport, traditional games, exercise, and brisk walking or cycling for recreation or to get to and from places, but should not include housework or physical activity that may be part of your job.

- 0
- 1
- 2
- 3
- 4
- 5
- 6
- 7

If >= 3 days/per week (achieving 90 mins of moderate-to-vigorous physical activity per week), procced to End of survey.

11. Do you anticipate a major absence (e.g., monthly long vacation, surgery) in the next 3 months?

- Yes (if selected, proceed to End of survey)
- No

12. Do you have a valid and active email address that you prefer to use to communicate with us and engage in this study?

- Yes (if selected, proceed to Q13)
- No (if selected, proceed to End of survey)

13. Please provide your email address:

Chapter 2: Patient Health Questionnaire, 9-item (PHQ-9)

| Over the last 2 weeks, how often have you been bothered by any of the following problems? | Not at all | Several days | More  than half the days | Nearly every day |
| --- | --- | --- | --- | --- |
| 1. Little interest or pleasure in doing things | 0 | 1 | 2 | 3 |
| 2. Feeling down, depressed, or hopeless | 0 | 1 | 2 | 3 |
| 3. Trouble falling or staying asleep, or sleeping too much | 0 | 1 | 2 | 3 |
| 4. Feeling tired or having little energy | 0 | 1 | 2 | 3 |
| 5. Poor appetite or overeating | 0 | 1 | 2 | 3 |
| 6. Feeling bad about yourself – or that you are a failure or have let yourself or your family down | 0 | 1 | 2 | 3 |
| 7. Trouble concentrating on things, such as reading the newspaper or watching television | 0 | 1 | 2 | 3 |
| 8. Moving or speaking so slowly that other people could have noticed? Or the opposite – being so fidgety or restless that you have been moving around a lot more than usual | 0 | 1 | 2 | 3 |
| 9. Thoughts that you would be better off dead or of hurting yourself in some way | 0 | 1 | 2 | 3 |

1. How difficult have these problems made it for you to do your work, take care of things at home, or get along with other people?

- Not difficult at all
- Somewhat difficult
- Very difficult
- Extremely difficult
