# Supplementary material for: Feasibility and preliminary effects of an app-based physical activity intervention for individuals with depression (MoodMover): A protocol for a single-arm, pre-post intervention study
Source: PLoS One. 2025 Apr 22;20(4):e0321958. doi: 10.1371/journal.pone.0321958 (PMC12013873; doi:10.1371/journal.pone.0321958)
Supplement: S4 File — (DOCX) [file pone.0321958.s004.docx]

**S4 File.** **Topics of major lessons**

| **Modules** | **Topics covered** |
| --- | --- |
| **1) Feeling better through daily activity** | - Exercise as a treatment option for depression - Mood and physical activity interaction - Physical benefits of exercise - Goal-setting |
| **2) Making physical activity enjoyable** | - Introducing affect - The importance of enjoying physical activity - Strategies and activities to increase the enjoyment of physical activity |
| **3) Building your self-confidence** | - Self-efficacy - How to increase self-efficacy - Exercise experiences shared by peers |
| **4) Building your physical activity opportunity** | - The influence of environment on behaviour - How to build environment for physical activity - Brainstorm physical activity opportunities - Grab and go activities |
| **5) Developing self-regulatory skills** | - Action planning - Coping planning |
| **6) Drawing on social support** | - Introducing social support - Build your social support - Thinking strategies for exercise - Positive self-talk |
| **7) Forming an exercise habit** | - Introducing habit - Relating habit to physical activity - How to form a habit (repetition, scripts, environmental cues) |
| **8) Building your exercise identity** | - Introducing WHO guidelines - Introducing exercise identity - Ways to increase exercise identity (commitment, enjoyment, social comparison, passion) |

*Note.* WHO = World Health Organization.
