# Supplementary material for: Feasibility and preliminary effects of an app-based physical activity intervention for individuals with depression (MoodMover): A protocol for a single-arm, pre-post intervention study
Source: PLoS One. 2025 Apr 22;20(4):e0321958. doi: 10.1371/journal.pone.0321958 (PMC12013873; doi:10.1371/journal.pone.0321958)
Supplement: S5 File — (DOCX) [file pone.0321958.s005.docx]

**S5 File.** **Demographic survey and clinical information.**

1. What is your age?
2. What is your height (m)?
3. What is your weight (kg)?
4. Please indicate your sex at birth.

- Female
- Male
- Prefer not to Answer

1. Please indicate your gender.

- Woman
- Man
- Non-binary person
- Prefer not to Answer

1. The Canadian Census identifies the following categories in its Census of the Population. Please indicate how you self-identify. This self-identification is not intended as an indication of one’s place of origin, citizenship, language or culture and recognizes that there are differences both between and among subgroups of persons of colour. If you are of mixed-descent, please indicate this by selecting all that apply, rather than using the “other” line unless parts of your self-identification do not appear in this list.

- Indigenous peoples of Canada
- Indigenous (outside of Canada)
- Arab
- Black
- Chinese (including Mainland China, Hong Kong, Macau and Taiwan)
- Filipino
- Japanese
- Korean
- Latin, Central, or South American (e.g., Brazilian, Chilean, Colombian, Mexican)
- South Asian (e.g., Indian, Pakistani, Sri Lankan, etc.)
- Southeast Asian (e.g., Cambodian, Indonesian, Laotian Vietnamese, etc.)
- West Asian (e.g., Afghan, Iranian, Syrian, etc.)
- White
- Other
- Prefer not to answer

1. Please indicate your ancestry here (if “Other” is selected to Q3).
2. What is the highest degree or level of education you have completed?

- Some high school
- High school
- Certificate or diploma
- Bachelor’s degree
- Master’s degree
- PhD
- Professional degree
- Prefer not to answer

1. What is your current employment status?

- Employed – part time (including self-employed)
- Employed – full time (including self-employed
- Stay-at-home parent
- Homemaker
- On maternity/parental leave
- On medical or disability
- Retired
- Unemployed
- Other
- Prefer not to answer

1. Please indicate your current employment status here (if “Other” is selected to Q6).
2. What is your before-tax household income?

- $0 - $19,999
- $20,000 - $39,999
- $40,000 - $59,999
- $60,000 - $79,999
- $80,000 - $99,999
- $100,000 - $119,999
- $120,000 - $139,999
- $140,000 or greater
- Prefer not to answer

1. Do you currently have a clinical diagnosis of major depressive disorder?

- Yes 🡪 13
- No 🡪 14

1. How long have you been diagnosed with depression?

- Less than 1 month
- 1-6 months
- 6-12 months
- 1-2 years
- More than 2 years

1. How long have you been experiencing symptoms of depression?

- Less than 1 month
- 1-6 months
- 6-12 months
- 1-2 years
- More than 2 years

1. Are you currently taking any medication for depression?

- Yes 🡪 16
- No 🡪 18

1. Please specify the name(s) and dosage(s) of the medication(s) you are currently taking:
2. How long have you been taking this/these medication(s)?

- Less than 1 month
- 1-6 months
- 6-12 months
- 1-2 years
- More than 2 years

1. Are you currently undergoing any other treatments for depression (e.g., psychotherapy, counseling, etc.)?

- Yes 🡪 19
- No 🡪 26

1. If yes, please specify the type(s) of treatment(s):
2. How long have you been receiving this/these treatment(s)?

- Less than 1 month
- 1-6 months
- 6-12 months
- 1-2 years
- More than 2 years

1. On a scale of 1 to 10, how effective do you find your current treatment for managing your depression symptoms? Scale from 1 (Not effective) to 10 (Very effective)
2. Have you noticed any side effects from your current treatment?

- Yes
- No

1. If yes, please specify the side effects:
2. Do you have any other ongoing medical conditions that are being treated along with your depression?

- Yes
- No

1. If yes, please specify the condition(s) and treatment(s):
2. Have you previously been treated for depression?

- Yes 🡪 25
- No 🡪 26

1. If yes, please specify the type(s) of previous treatment(s) and duration:

**Alcohol Use (From CCWS)**

For the purpose of this survey, a drink means:

• 341 ml or 12 oz. of beer or cooler (bottle, can, or draft)

• 142 ml or 5 oz. of wine

• 43 ml or 1.5 oz. of liquor or spirit (straight or mixed)

Include light beer.

Exclude de-alcoholised beer or coolers (0.5% alcohol) or cocktails such as Virgin Mary or Shirley Temple.

The physiological effects of alcohol vary by biological sex. For this question, refer to the number of drinks that corresponds with your biological sex at birth.

1. During the past 30 days, how often have you had 4 or more drinks (female sex) OR 5 or more drinks (male sex) on one occasion? *“On one occasion” means at the same time or within a couple of hours of each other.*

- Daily or almost daily
- 2 to 5 times a week
- Once a week
- 2 to 3 times in the past 30 days
- Once in the past 30 days
- Not in the past 30 days
- I do not drink alcohol
- I don't know
- I prefer not to answer

**Cannabis Use (From CCWS)**

The next questions are about **cannabis**. In this survey when we use the term cannabis, this includes **marijuana (e.g., weed, pot), hashish, hash oil or any other products made from the cannabis plant, but not synthetic cannabinoids.**

When we ask about use, this includes using cannabis in its dry form or when mixed or processed into another product such as an edible, an extract, a concentrate, including hashish, a liquid, or other product.

Cannabis use may include use for medical and/or non-medical purposes.

1. In the past 12 months, have you used cannabis?

- Yes 🡪 30
- No
- I don’t know
- I prefer not to answer

1. If yes, in the past 30 days, how often did you use cannabis?

- Not in the past 30 days
- 1 day in the past 30 days
- 2 or 3 days in the past 30 days
- 1 or 2 day(s) per week
- 3 or 4 days per week
- 5 or 6 days per week
- Daily
- I don't know
- I prefer not to answer

1. Are you currently using any other physical activity apps or devices (e.g., Fitbit, Garmin, Apple Watch)?

o Yes 🡪 32

o No 🡪 33

1. How frequently do you use these physical activity apps or devices?

o Not in the past 30 days

o 1 day in the past 30 days

o 2 or 3 days in the past 30 days

o 1 or 2 day(s) per week

o 3 or 4 days per week

o 5 or 6 days per week

o Daily

o I don't know

o I prefer not to answer

1. Have you ever used any physical activity apps or devices in the past?

o Yes 🡪 34

o No

1. For how long did you use these physical activity apps or devices?

- Less than 1 week
- 2-4 weeks
- 1-2 months
- 2-3 months
- 3-4 months
- 4-6 months
- Over 6 months
