# Supplementary material for: Feasibility and preliminary effects of an app-based physical activity intervention for individuals with depression (MoodMover): A protocol for a single-arm, pre-post intervention study
Source: PLoS One. 2025 Apr 22;20(4):e0321958. doi: 10.1371/journal.pone.0321958 (PMC12013873; doi:10.1371/journal.pone.0321958)
Supplement: S7 File — (DOCX) [file pone.0321958.s007.docx]

**S7 File. Satisfaction**

1. **The mHealth Satisfaction Questionnaire, 14-item**

|  | ***Strongly Disagree***  *1* | *2* | *3* | *4* | ***Strongly Agree***  *5* |
| --- | --- | --- | --- | --- | --- |
| **What did you think about using the health app?** | | | | | |
| It was easy to use |  |  |  |  |  |
| It was good to use |  |  |  |  |  |
| The time spent using it has been acceptable |  |  |  |  |  |
| It has been difficult to remember to use it |  |  |  |  |  |
| The introduction of how to use it was sufficient |  |  |  |  |  |
| It was too time consuming |  |  |  |  |  |
| It interrupted me in my daily activities |  |  |  |  |  |
| It was boring to use |  |  |  |  |  |
| It was a disturbance |  |  |  |  |  |
| I can recommend it to others |  |  |  |  |  |
| **How did you experience the health app?** | | | | | |
| It has motivated me to change my lifestyle habits |  |  |  |  |  |
| It has helped me to understand the benefits of improving my lifestyle habits |  |  |  |  |  |
| It has helped me to understand how I need to change my lifestyle habits |  |  |  |  |  |
| It has helped me set personal goals for my lifestyle habits in a way that I could not have done on my own |  |  |  |  |  |

1. **Please list three aspects of the program/MoodMover that you particularly liked and three aspects that you disliked. You may refer to things such as the content, delivery, or support.**
