# Supplementary material for: Feasibility and preliminary effects of an app-based physical activity intervention for individuals with depression (MoodMover): A protocol for a single-arm, pre-post intervention study
Source: PLoS One. 2025 Apr 22;20(4):e0321958. doi: 10.1371/journal.pone.0321958 (PMC12013873; doi:10.1371/journal.pone.0321958)
Supplement: S8 File — (DOCX) [file pone.0321958.s008.docx]

**S8 File. Physical Activity Adult Questionnaire (PAAQ)**

Assuming the interview is conducted on a **Monday**.

Answer categories are provided in parentheses.

The following questions are about various types of physical activities done in the last 7 days.

1. In the last 7 days, that is from last Sunday to yesterday, did you use active ways like walking or cycling to get to places such as work, school, the bus stop, the shopping centre or to visit friends (active transportation)?

(Yes or No)

2. In the last 7 days, on which days did you do these activities?

(Monday through Sunday)

3. How much time in total, in the last 7 days, did you spend doing these activities?

(Type in answer)

4. In the last 7 days, did you do sports, fitness or recreational physical activities, organized or non-organized? Examples are walking, home or gym exercise, swimming, cycling, running, skiing, dancing and all team sports.

(Yes or No)

5. Did any of these recreational physical activities make you sweat at least a little and breathe harder?

(Yes or No)

6. In the last 7 days, on which days did you do these recreational activities that made you sweat at least a little and breathe harder?

(Monday through Sunday)

7. In the last 7 days, how much time in total did you spend doing these activities that made you sweat at least a little and breathe harder?

(Type in answer)

8. In the last 7 days, did you do any other physical activities while at work, in or around your home or while volunteering? Examples are carrying heavy loads, shoveling, and household chores such as vacuuming or washing windows. (Yes or No)

9. Did any of these other physical activities make you sweat at least a little and breathe harder?

(Yes or No)

10. In the last 7 days, on which days did you do these other activities that made you sweat at least a little and breathe harder?

(Monday through Sunday)

11. In the last 7 days, how much time in total did you spend doing these activities that made you sweat at least a little and breathe harder?

(Type in answer)

12. If you have reported any minutes of physical activity that made you sweat at least a little and breathe harder. Of these activities, were there any of vigorous intensity, meaning they caused you to be out of breath?

(Yes or No)

13. In the last 7 days, how much time **in total** did you spend doing vigorous activities that caused you to be out of breath?

(Type in answer)

14. During the program, did you link a smartwatch or fitness device to the Health app (iOS) or Google Fit (Android)? **–[post-intervention only]**

(Yes or No)

15. How often did you carry your phone or wear your smartwatch during your non-sedentary waking hours on workdays? **–[post-intervention only]**

(Almost always, Sometimes, Seldom)

16. How often did you carry your phone or wear your smartwatch during your non-sedentary waking hours on weekends? **–[post-intervention only]**

(Almost always, Sometimes, Seldom)
