# Supplementary material for: Feasibility and preliminary effects of an app-based physical activity intervention for individuals with depression (MoodMover): A protocol for a single-arm, pre-post intervention study
Source: PLoS One. 2025 Apr 22;20(4):e0321958. doi: 10.1371/journal.pone.0321958 (PMC12013873; doi:10.1371/journal.pone.0321958)
Supplement: S10 File — (DOCX) [file pone.0321958.s010.docx]

**S10 File. The Pittsburgh Sleep Quality Index (PSQI)**

Instructions: The following questions relate to your usual sleep habits during **the past month only**. Your answers indicate the most accurate reply for the **majority** of days and nights in the past month. **Please answer all the questions.**

During the past month,

1. When have you usually gone to bed? _________________

2. How long (in minutes) has it taken you to fall asleep each night? ________________

3. When have you usually gotten up in the morning? _________________

4. How many hours of actual sleep do you get at night? (This may be different than the numbers of hours you spend in bed) _________________

| 5. During the past month, how often have you had trouble sleeping because you… | Not during the past month (0) | Less than once a week (1) | Once or twice a week (2) | Three or more times a week (3) |
| --- | --- | --- | --- | --- |
| 1. Cannot get to sleep within 30 minutes |  |  |  |  |
| 1. Wake up in the middle of the night or early morning |  |  |  |  |
| 1. Have to get up to use the bathroom |  |  |  |  |
| 1. Cannot breathe comfortably |  |  |  |  |
| 1. Cough or snore loudly |  |  |  |  |
| 1. Feel too cold |  |  |  |  |
| 1. Feel too hot |  |  |  |  |
| 1. Have bad dreams |  |  |  |  |
| 1. Have pain |  |  |  |  |
| 1. Other reason(s), please describe, including how often you have had trouble sleeping because of this reason(s): |  |  |  |  |
| 6. During the past month, how often have you taken medicine (prescribed or “over the counter”) to help you sleep? |  |  |  |  |
| 7. During the past month, how often have you had trouble staying awake while driving, eating meals, or engaging in social activity? |  |  |  |  |
| 8. During the past month, how much of a problem has it been for you to keep up enthusiasm to get things done? |  |  |  |  |
|  | Very good (0) | Fairly good (1) | Fairly bad (2) | Very bad (3) |
| 9. During the past month, how would you rate your sleep quality overall? |  |  |  |  |

**Scoring the PSQI**

In scoring the PSQI, seven component scores are derived, each scored 0 (no difficulty) to 3 (severe difficulty). The component scores are summed to produce a global score (range 0 to 21). Higher scores indicate worse sleep quality.

**Component 1:** **Subjective sleep quality—question 9**

#9 Score **. . . . . . . . . . . . . . . . . . . . . . . . . . . . . . . . . . . . . . . . . . . . . . . . . . . .** C1______

**Component 2: Sleep latency—questions 2 and 5a**

#2 Score (≤15min=0; 16-30 min=1; 31-60 min=2; >60 min=3) +#5a Score

(if sum is equal 0=0; 1-2=1; 3-4=2; 5-6=3**) . . . . . . . . . . . . . . . . . . . . . . . . .** C2______

**Component 3: Sleep duration—question 4**

#4 Score (>7=0; 6-7=1; 5-6=2; <5=3**) . . . . . . . . . . . . . . . . . . . . . . . . . . . . .** C3______

**Component 4: Sleep efficiency—questions 1, 3, and 4**

(total # of hours asleep)/(total # of hours in bed) x 100

>85%=0, 75%-84%=1, 65%-74%=2, <65%=3 **. . . . . . . . . . . . . . . . . . . . . .**  C4______

**Component 5: Sleep disturbance—questions 5b-5j**

Sum of scores #5b to #5j (0=0; 1-9=1; 10-18=2; 19-27=3**) . . . . . . . . . . . . .** C5______

**Component 6: Use of sleep medication—question 6**

#6 Score **. . . . . . . . . . . . . . . . . . . . . . . . . . . . . . . . . . . . . . . . . . . . . . . . . . . .** C6______

**Component 7: Daytime dysfunction**

#7 Score + #8 Score (0=0; 1-2=1; 3-4=2; 5-6=3) **. . . . . . . . . . . . . . . . . . . . .** C7______

Add the seven component scores together______ **Global PSQI Score**_______

Citation: Buysse, DJ, Reynolds CF, Monk TH, Berman SR, Kupfer DJ: The Pittsburgh

Sleep Quality Index (PSQI): A new instrument for psychiatric research and

practice. Psychiatry Research 28:193-213, 1989
