# Supplementary material for: Feasibility and preliminary effects of an app-based physical activity intervention for individuals with depression (MoodMover): A protocol for a single-arm, pre-post intervention study
Source: PLoS One. 2025 Apr 22;20(4):e0321958. doi: 10.1371/journal.pone.0321958 (PMC12013873; doi:10.1371/journal.pone.0321958)
Supplement: S11 File — (DOCX) [file pone.0321958.s011.docx]

**S11 File. WHODAS 2.0 12-item version, self-administered**

This questionnaire asks about difficulties due to health conditions. Health conditions include diseases or illnesses, other health problems that may be short or long lasting, injuries, mental or emotional problems, and problems with alcohol or drugs.

Think back over the past 30 days and answer these questions, thinking about how much difficulty you had doing the following activities. For each question, please circle only one response.

| In the past 30 days, how much difficulty did you have in: | | | | | | |
| --- | --- | --- | --- | --- | --- | --- |
| S1 | Standing for long periods such as 30 minutes? | None | Mild | Moderate | Severe | Extreme or cannot do |
| S2 | Taking care of your household responsibilities? | None | Mild | Moderate | Severe | Extreme or cannot do |
| S3 | Learning a new task, for example, learning how to get to a new place? | None | Mild | Moderate | Severe | Extreme or cannot do |
| S4 | How much of a problem did you have joining in community activities (for example, festivities, religious or other activities) in the same way as anyone else can? | None | Mild | Moderate | Severe | Extreme or cannot do |
| S5 | How much have you been emotionally affected by your health problems? | None | Mild | Moderate | Severe | Extreme or cannot do |

***Please continue to next page...***

| In the past 30 days, how much difficulty did you have in: | | | | | | |
| --- | --- | --- | --- | --- | --- | --- |
| S6 | Concentrating on doing something for ten minutes? | None | Mild | Moderate | Severe | Extreme or cannot do |
| S7 | Walking a long distance such as a kilometre [or equivalent]? | None | Mild | Moderate | Severe | Extreme or cannot do |
| S8 | Washing your whole body? | None | Mild | Moderate | Severe | Extreme or cannot do |
| S9 | Getting dressed? | None | Mild | Moderate | Severe | Extreme or cannot do |
| S10 | Dealing with people you do not know? | None | Mild | Moderate | Severe | Extreme or cannot do |
| S11 | Maintaining a friendship? | None | Mild | Moderate | Severe | Extreme or cannot do |
| S12 | Your day-to-day work? | None | Mild | Moderate | Severe | Extreme or cannot do |

| H1 | Overall, in the past 30 days, how many days were these difficulties present? | ***Record number of days*** |
| --- | --- | --- |
| H2 | In the past 30 days, for how many days were you totally unable to carry out your usual activities or work because of any health condition? | ***Record number of days*** |
| H3 | In the past 30 days, not counting the days that you were totally unable, for how many days did you cut back or reduce your usual activities or work because of any health condition? | ***Record number of days*** |

This completes the questionnaire. Thank you.
