# Supplementary material for: Feasibility and preliminary effects of an app-based physical activity intervention for individuals with depression (MoodMover): A protocol for a single-arm, pre-post intervention study
Source: PLoS One. 2025 Apr 22;20(4):e0321958. doi: 10.1371/journal.pone.0321958 (PMC12013873; doi:10.1371/journal.pone.0321958)
Supplement: S12 File — (DOCX) [file pone.0321958.s012.docx]

**S12 File. M-PAC constructs**

**Physical Activity**

The next questions ask about your thoughts and feelings about physical activity. Before we begin, please take an extra minute to read this section. It is very important that you understand what we mean when we say ***physical activity.***

***Physical activity*** is movement that takes effort and makes your heart beat faster. You might start to breathe harder, feel warm or flushed, or sweat.

Examples of physical activity include brisk walking, swimming, tennis, cycling, basketball, hiking, or paddle boarding

**ATTITUDES TOWARD PHYSICAL ACTIVITY**

***Affective Attitudes*** (Rhodes et al., 2010)

For me, participating in regular physical activity over the next month would be:

*(Responses on a 1 to 7 scale)*

1. Unenjoyable to enjoyable
2. Boring to very exciting
3. Unpleasant to very pleasant

***Instrumental Attitudes*** (Rhodes et al., 2010)

For me, participating in regular physical activity over the next month would be:

*(Responses on a 1 to 7 scale)*

1. Unwise to very wise
2. Not beneficial to very beneficial
3. Useless to very useful

**PERCEIVED CAPABILITY** (Rhodes et al., 2006)

These questions ask you about your confidence and/or control over engaging in regular physical activity. Please select a number for each question using the scale provided.

Consider what you would do for each question, assuming you wanted to do the task.

Please indicate your level of agreement with the following statements:

*(Responses on a 1 to 5, Strongly Disagree to Strongly Agree, scale)*

1. I have the skills I need to be physically active
2. I am physically capable of doing moderate to vigorous physical activity for 150 min across a week if I really had to
3. I am confident in my ability to engage in physical activity.

**PERCEIVED OPPORTUNITY FOR PHYSICAL ACTIVITY** (Rhodes et al, 2006)

These questions ask you about your opportunity for engaging in regular exercise. Please circle a number for each question using the scale provided.

*(Responses on a 1 to 5, Strongly Disagree to Strongly Agree, scale)*

1. I have the opportunity to be physically active every week if I really had to
2. I will have opportunities to increase my physical activity over the next month
3. I will have opportunities to track my physical activities via the app over the next month.

**INTENTION STRENGTH TO BE PHYSICALLY ACTIVE** (Rhodes et al., 2010)

These questions ask you about your intention for engaging in regular exercise. Please circle a number for each question using the scale provided.

*(Responses on a 1 to 5, Strongly Disagree to Strongly Agree, scale)*

1. I am committed to engage in physical activity over the next month
2. I am motivated to engage in PA over the next month
3. In the next 6 months, I have intentions to be physically active regularly

**DECISIONAL INTENTIONS TO BE PHYSICALLY ACTIVE**

The following will ask you about your intention to be regularly physically active.

I intend to increase 3000 steps above my baseline daily steps for most days per week. (Yes/No)

**BEHAVIOURAL REGULATION FOR PHYSICAL ACTIVITY** (Rovniak et al. 2002)

Sometimes we use strategies to help us to be physically active. Please use the following scale to answer the questions below.

*(Responses on a 1 to 5, Strongly Disagree to Strongly Agree, scale)*

1. I often monitor how physically active I am
2. I often set physical activity goals
3. I often plan, when, what, where, and how I am going to be physically active

**HABIT OF PHYSICAL ACTIVITY** (Gardner, Abraham, Lally, & de Bruijn, 2012; Rhodes and Lim, 2016)

The following questions ask about your habits regarding physical activity. Habits are behaviors that are so practiced they are often done without much thought or motivation. For example, for many people driving a car becomes a habit when one can think about all sorts of things, concentrate on the road, and give little thought to operating the controls of the car. Sometimes travelling to and from work becomes such a habit that we do not even think about our route anymore. Please select the answer that best represents you.

*(Responses on a 1 to 5, Strongly Disagree to Strongly Agree, scale)*

1. I engage in regular physical activity without having to consciously remember it
2. I engage in regular physical activity automatically
3. I engage in regular physical activity without consciously thinking about it

**PHYSICAL ACTIVITY IDENTITY** (Wilson & Muon, 2008; Rhodes and Lim, 2016)

The following questions concern your personal beliefs about exercise. Please indicate the degree to which you agree or disagree with each statement when thinking about your exercise participation.

*(Responses on a 1 to 5, Strongly Disagree to Strongly Agree, scale)*

1. I consider myself someone who is physically active
2. When I describe myself to others, I usually include my involvement in physical activity
3. Others see me as someone who does physical activity regularly
